# Clinician-driven automated data preprocessing in nuclear medicine AI environments

**DOI:** 10.1007/s00259-025-07183-5

**Published:** 2025-03-07

**Authors:** Denis Krajnc, Clemens P. Spielvogel, Boglarka Ecsedi, Zsombor Ritter, H. Alizadeh, Marcus Hacker, Laszlo Papp

**Affiliations:** 1 Center for Medical Physics and Biomedical Engineering, Medical University of Vienna, Vienna, Austria; 2 Department of Biomedical Imaging and Image-guided Therapy, Division of Nuclear Medicine, Medical University of Vienna, Vienna, Austria; 3 Georgia Institute of Technology, Atlanta, GA USA; 4 Department of Medical Imaging, Medical School, University of Pécs, Pécs, Hungary; 5 Medical School, 1st Department of Internal Medicine, University of Pécs, Pécs, Hungary; 6 Applied Quantum Computing Group, Center for Medical Physics and Biomedical Engineering, Medical University of Vienna, Währinger Gürtel 18- 20, Vienna, 1090 Austria

**Keywords:** AI, Data preprocessing, Cancer, Imaging analysis

## Abstract

**Background:**

Artificial Intelligence (AI) approaches in clinical science require extensive data preprocessing (DP) steps prior to building AI models. Establishing DP pipelines is a non-trivial task, mainly driven by purely mathematical rules and done by data scientists. Nevertheless, clinician presence shall be paramount at this step. The study proposes a data preprocessing approach driven by clinical domain knowledge, where clinician input, in form of explicit and non-explicit rules, directly impacts the algorithms’ decision-making processes, thus, making the DP planning phase more inclusive for clinicians.

**Methods:**

The rule set table (RST) was introduced as interface which accepts clinician’s input as formal rules (including four actions: exp-keep, exp-remove, pref-keep, pref-remove features or samples) in human-readable form and translates it to machine readable input for preprocessing algorithms. A collection of commonly used algorithms was incorporated for data preprocessing of various clinical cohorts in both single and multi-center scenarios. The impact of RST was evaluated by utilizing 100-fold Monte Carlo cross-validation scheme for prostate and glioma cohorts (single center) with 80 − 20% training-testing split. Furthermore, diffuse large B-cell lymphoma (DLBCL) cohort was evaluated by using Center 1 as training and Center 2 as testing cohort for clinical endpoint prediction. Both scenarios were investigated in manual and automated data preprocessing setups across all cohorts. The XGBoost algorithm was employed for classification tasks across all established models. Predictive performance was estimated by confusion matrix analysis in validation samples of all cohorts. The performance of RST across all actions as well as without RST were compared in both manual and automated settings for each respective cohort.

**Results:**

Performance increase of ML models with manual preprocessing combined with RST was up-to 18% balanced accuracy (BACC) compared to models without RST. The ML models with “exp-keep” and “pref-keep” instructions showed highest performance increase of + 18% BACC (glioma), + 6% BACC (prostate) and + 3% BACC (DLBCL) compared to other models across all datasets.

**Conclusion:**

The study demonstrated the added value of RST in predictive performance of oncology-specific ML models, hence, serving as proof of concept of a more inclusive clinician-driven DP process in future studies.

**Supplementary Information:**

The online version contains supplementary material available at 10.1007/s00259-025-07183-5.

## Introduction

In recent years, we witnessed the continuous involvement of artificial intelligence (AI) in the field of medical image analysis [1], where different machine learning (ML) approaches such as traditional ML or deep learning based on complex neural networks, were recognized as promising technology for non-invasive disease characterization and classification tasks [2]. Radiomic analysis combined with ML methods demonstrated high potential in building predictive models for numerous cancer diseases [3–5], especially in cases of small patient cohorts, where large-scale data collection is difficult to achieve due to various limitations [6, 7]. Data preprocessing became an integral part of hybrid imaging ML analysis, where an abundant number of studies incorporated such methods as an essential step in the model training pipeline [8–10]. Since this process requires a significant amount of data scientist expertise, it is itself a very subjective activity, thus, it is difficult to standardize [11, 12]. Due to its high complexity and esoteric nature, it had been proposed that data preprocessing itself should be automatized to achieve optimal ML training performance [12]. In contrast to focusing only on data preprocessing, commercial AutoML solutions are available, that fully automatize ML classifier building and selection of best-performing models as well as identifying their respective hyperparameters that influence the process of ML training. However, the effects of data preprocessing is not possible to solely assess in such systems, as data preprocessing steps are often also guided by hyperparameter identification steps [13]. Nevertheless, all current available solutions are purely based on complex mathematical methodologies [14], hence clinical domain experts without in-depth AI knowledge have little to no opportunities to directly interact with data preprocessing algorithms. Such interaction could potentially be highly important, given that clinicians may possess specific knowledge about the disease which could lead to improved training procedures of ML models.

With this being considered, this study aims to implement a novel data preprocessing approach which incorporates expert domain inputs in the data preprocessing process in cancer imaging AI settings. The objectives of this study are: (a) to implement a data preprocessing approach which incorporates domain expert directives in a human-written form, throughout a rule-set table (RST) interpretation; (b) to collect clinically relevant patient datasets, which will support the testing of the previously mentioned method in single-center cross validation and dual-center independent test scenarios; and (c) to estimate the effectiveness of RST in various scenarios such as involving ML-driven data preprocessing (MLDP) with RST, manually predefined fixed data preprocessing pipeline with RST and MLDP without RST.

## Methods

### Cohorts

Three patient cohorts with different cancer disease were analyzed in total. The data collection for glioma and prostate cancer patients originated from single centers were analysis to predict 36-months survival and high-vs-low risk clinical endpoints respectively [15, 16], and DLBCL data was acquired from two different hospitals, to predict the 24-months progression [17].

For glioma cancer cohort, patients (*n* = 69) with histologically verified treatment-naïve were collected. The cohort included only immunohistochemical staining verified amino acid–positive cases with a known IHD1 R132H mutational status [15]. The DLBCL cohort was collected in two independent centers (*n* = 41 and *n* = 44), where patients with baseline pretreatment were considered. The treatment underwent a standard R-CHOP-21 treatment regimen for at least 4 full cycles [17]. The patients were clustered according to germinal center B-cell-like (GCB) or activated B-cell (non-GCB) type using the Hans algorithm [17]. Prostate cohort included patients with primary prostate cancer (*n* = 57) who underwent multi-parametric dual-tracer [18 F]FMC and [68Ga]Ga-PSMA-11 PET/MRI as well as radical prostatectomy [16]. See the respective references [15–17] of involved data collection for detailed description of incorporated datasets and their respective Imaging Biomarker Standardization Initiative (IBSI)-conform extracted radiomic features.

**Fig. 1:**
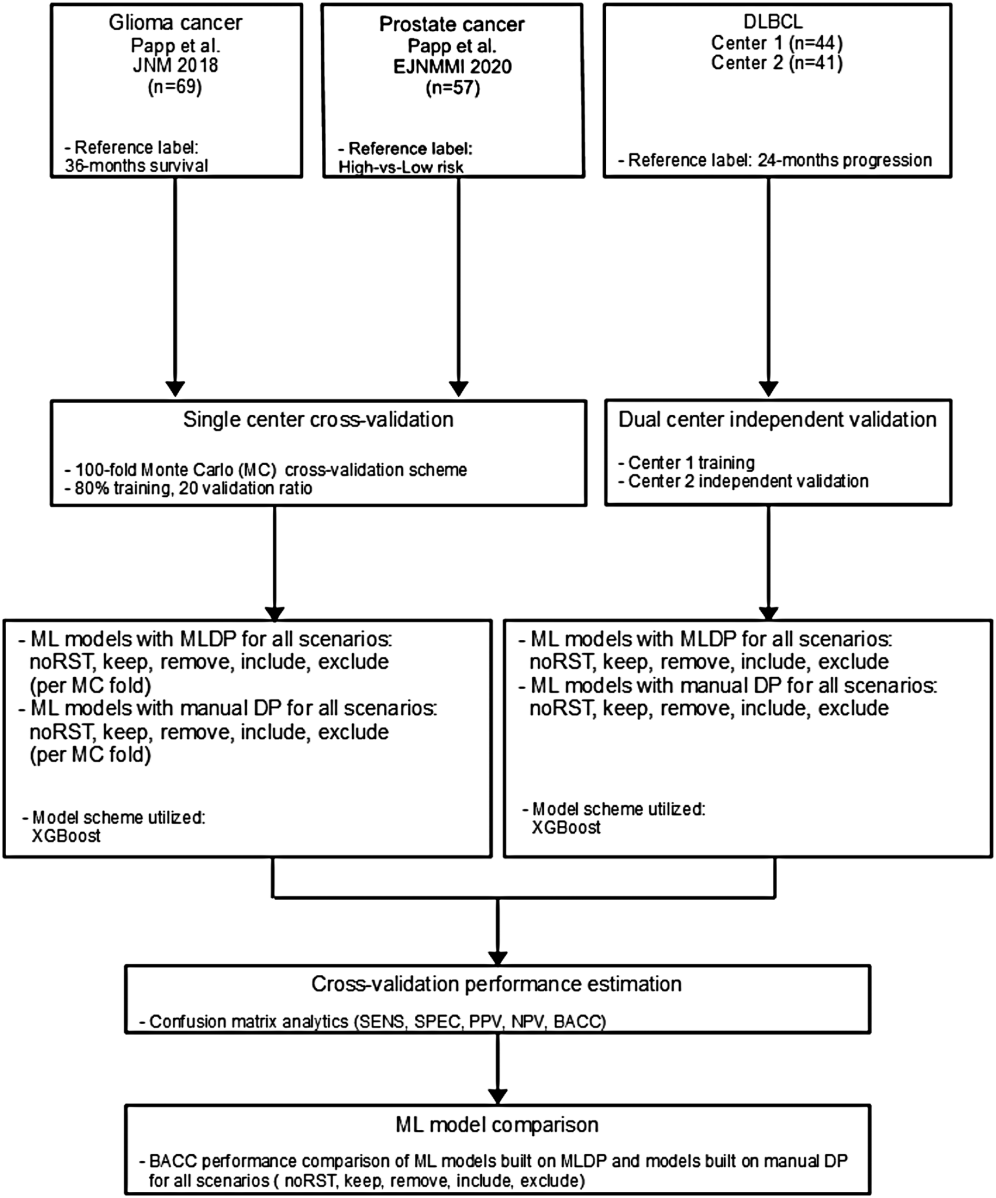
In this study, two single center glioma and prostate cancer and one dual-center diffuse large B-cell lymphoma (DLBCL) cohorts were analyzed retrospectively. For single center data, 100-fold Monte Carlo (MC) cross-validation scheme was utilized with 80%-20% training/validation data split. For dual center DLBCL analysis, Center 1 dataset was used for training and Center 2 for independent validation. Predictive models were established with and without machine learning-driven data preparation (MLDP) per training-validation pair in each cohort, where both scenarios included RST directives (exp-keep, exp-remove, pref-keep, pref-remove) as well as model training without RST. All built models utilizing the Extreme gradient boosting (XGBoost) learning scheme. Predictive performance of each model scheme was evaluated with confusion matrix analytics. Performance comparison of ML models with and without incorporated MLDP was conducted for each analyzed cohort. DLBCL – Diffuse large B-cell lymphoma; ACC – Accuracy; SNS – Sensitivity; SPC – Specificity; PPV – Positive predictive value; NPV – Negative predictive value; BACC – Balanced accuracy

The datasets used in this study (Table 1) were selected with respect to their non-similarity in terms of patient count, dimensionality, subclass imbalance ratio and data origin (single vs. dual-center). See Fig. 1 for the overall flow of the proposed study. See Supplemental Table S8 for the CLAIM AI reporting guideline.

**Table 1:** Characteristics of cancer cohorts used in study. MET – Methionine; PET – Positron emission tomography; FNA – Fine needle aspiration; PSMA – Prostate specific membrane antigen; MRI – Magnetic resonance imaging; CT – Computed tomography; DLBCL – Diffuse large B-cell lymphoma. Table is from [12]

Cohort	Prediction	Centers	Data	Samples	Features	Imbalance ratio [%]	Reference
Glioma	36-months survival	Single	^11^C-MET PET	69	160	67-vs-33	[15]
Prostate cancer	high-vs-low risk	Single	^68^GA-PSMA PET/MRI	57	306	52-vs-48	[16]
DLBCL Center 1	24-months progression	Dual	^18^F-FDG PET/CT	44	57	32-vs-68	[17]
DLBCL Center 2				41		39-vs-61	

### Feature extraction

The feature extraction was performed in line with the Imaging Biomarker Standardization Initiative (IBSI), where features with both “strong” and “very strong” consensus were extracted for each cancer cohort to enable the reproducibility of the study [18]. The extracted features were combined with additional patient demographics information for all three cohorts (DLBCL: gender and age at diagnosis; Glioma: age, weight, height, Karnofsky score, WHO 2007 grade, histology and IDH1 R132H mutation status; Prostate: age, height, weight) to form a holomics dataset [15] for further ML analysis. The feature extraction process was executed by the in-house MUW Radiomics engine (v2.0) validated according to IBSI standards [18].

### Data preprocessing with rule set tables

**Fig. 2:**
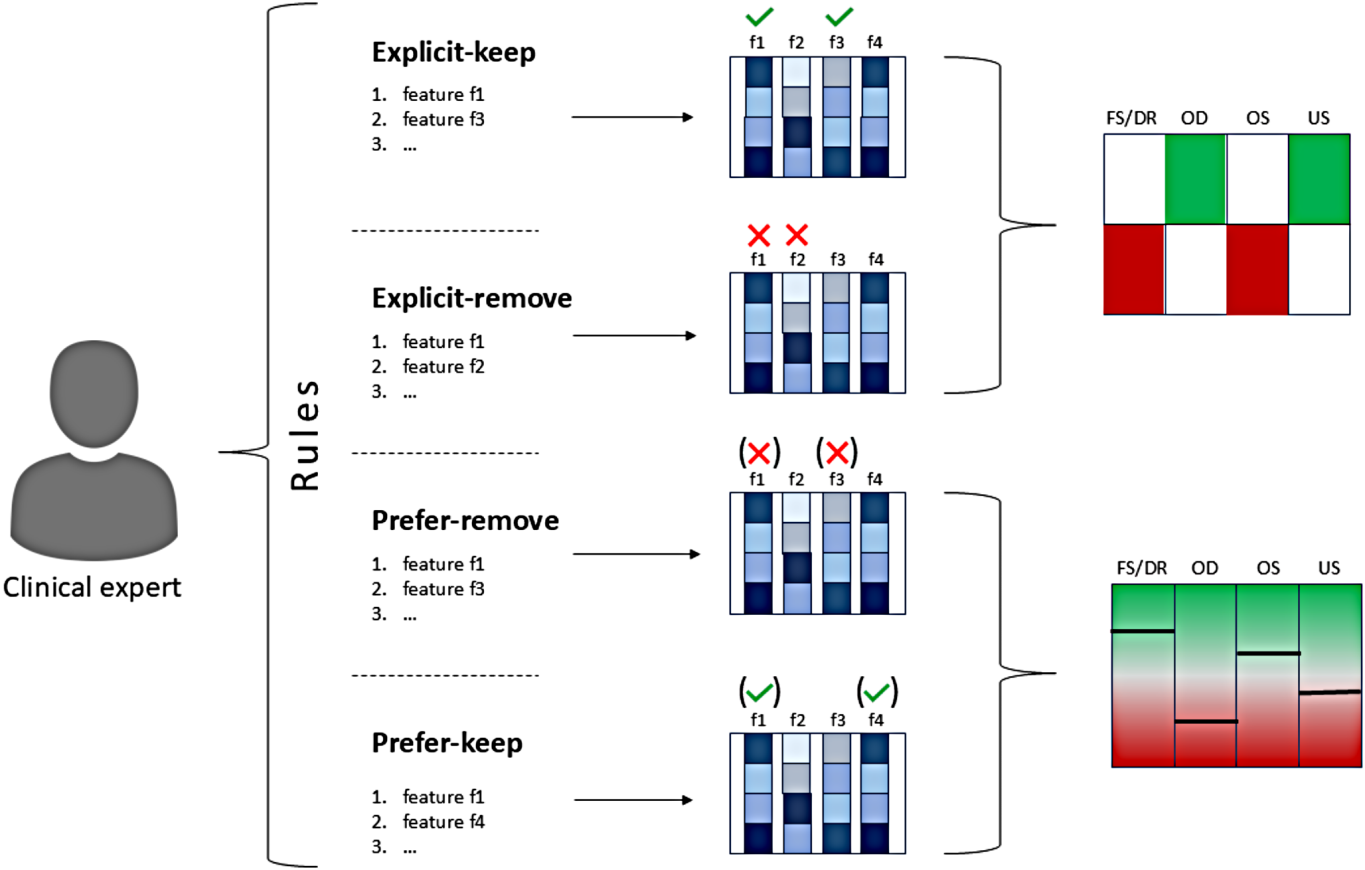
The workflow of rule set table (RST): The RST allows clinical experts to affect the data preprocessing phase by providing definite rules (exp-keep, exp-remove), where features/samples will be kept/removed regardless of the decisions of preprocessing algorithms. In addition, clinical experts can provide non-definite rules or “suggestions” in form of the pref-keep/pref-remove directives, where features/samples are preferred to be kept/removed, however the final decision is performed within the algorithm itself, based algorithm-specific criteria (Supplemental Table S1)

In order to enable direct involvement of clinical experts in the ML decision making process, the RST is introduced as interface for communication between the clinicians and data preprocessing algorithms. The main motivation for the clinical use of such tool is to influence and gain a certain portion of control over the data preprocessing processes. The interaction between the clinician and the RST method is straightforward: clinician is editing desired rules in a text file, following the simple predefined syntax (Supplemental S9). The RST accepts instructions in the form of formal rules as an input, which specifies the desired action to influence DP steps. The actions are categorized into four categories: explicit keep (exp-keep), explicit remove (exp-remove), prefer keep (pref-keep), prefer remove (pref-remove). The exp-keep and exp-remove actions are explicit instructions whether a certain feature or sample will be kept or removed, regardless of the data preprocessing algorithm decision, thus it is a hard-requirement from the clinician to follow. In contrast, the pref-keep and pref-remove actions allow the clinician to generate preferential rules. These preferential rules are suggestions to data preprocessing algorithms that can consider these preferences when filtering out samples or features. When such decisions are made the given data preprocessing algorithm can consider giving priority to samples or features to pref-keep/pref-remove them based on the given rule, in case the given sample or feature are ranked as high-ranking to perform the given action, even if they are not the highest-ranking ones. Therefore, the main difference between exp-keep - exp-remove and pref-keep - pref-remove is that latter ones can still be overruled by the given method if the suggestion would result in suboptimal performance (a.k.a. the given sample or feature are too low-ranking to perform the given action) (Fig. 2). The decision-making criteria are designed specifically for each preprocessing algorithm (See Supplemental Table S1 for detailed description of respective individual criteria). In this study, paradigms with all four (exp-keep, exp-remove, pref-keep, pref-remove) directives are tested per each cohort to demonstrate the functionality of the RST approach, however, in the real-world scenario, single or multiple rules of various types (e.g. conditional rules) are supported to guide the preprocessing system (See Supplemental Table S9 for the example of various rules).

### Data preprocessing pipelines

For each patient cohort, two data preprocessing approaches were utilized: manually fixed data preprocessing pipeline (manual DP) and machine learning-driven data preprocessing (MLDP) [12]. The manual DP was assembled of the synthetic minority oversampling technique (SMOTE) algorithm [19] for data imbalance correction, isolation forest algorithm [20] for outlier detection and sequential forward selection (SFS) [21] for feature selection. Each algorithm had fixed values of respective hyperparameters, which were chosen based on best practices and guidelines according to the corresponding literature (Supplemental Table S2). The algorithm selection for the preprocessing pipeline was created based on the literature review of similar works in which the aforementioned algorithms were represented as the most commonly utilized approaches [22–24]. The SFS-based feature selection method was incorporated in both MLDP (if included by the system) and manual preprocessing pipelines, where the subset of selected features in MLDP was determined by the system itself. For the manual approach, the subset of features was in alignment with previous works (Glioma = 20 features; Prostate = 20 features; DLBCL = 10 features) [12, 15–17]. The manual preprocessing approach was evaluated to ensure the estimation of RST effects on the training procedures.

Next to the manual DP, the MLDP approach was employed as an automated ML-driven data preprocessing solution, to generate the optimal pipeline of data preprocessing algorithms combined with its automated hyperparameter optimization steps. For both approaches, five possible rule scenarios were established: no rules (data preprocessing is not affected by clinical expert), exp-keep, exp-remove, pref-keep, pref-remove (see chapter Data Preprocessing with Rule Set Tables for detailed explanation of incorporated rules). All rule scenarios combined with data preprocessing approaches were applied across all analyzed patient cohorts. For conducting comparative analyses, and to ensure the strong reference point for RST method validation, exp-keep and exp-remove rules focused on the five highest-ranking features per-cohort, as identified in prior reports [15–17]. The above approach resulted in 10 experiments per-cohort (manual DP and MLDP with 5 rule scenarios each).

### Feature analysis

To analyze the feature presence in the trained ML models, the overall feature occurrence is calculated across all folds for each individual built model per cohort. The incorporated features are further compared across ML models with included directives per cohort to identify the effects of external domain input on the predictive performance. For ML models built over the manually preprocessed data, the feature count was determined with respect to the “curse of dimensionality” paradigm [25].

### ML model training

To build predictive models per-cohort and per-experiment, the extreme gradient boosting (XGBoost) [26], random forest (RF) and supported vector machine (SVM) algorithms were employed. For both data preprocessing approaches (manual and MLDP) combined with all rule scenarios, the predictive models were trained for each patient cohort, which resulted in 30 established ML models.

Performance Evaluation.

To evaluate the performance of ML models, a 100-fold Monte Carlo cross-validation (MCCV) scheme with the 80% − 20% train-test split ratio was utilized for single-center (glioma and prostate cancer) datasets. The split configurations were maintained across all 10 experiments per single-center cohort to eliminate ML test performance variations related to repeated fold split randomities. In case of dual-center DLBCL dataset, Center 1 was used for training and Center 2 for performance validation. The performance value calculations were done by test Confusion matrix [12] analysis, which yielded metrics such as sensitivity (SENS), specificity (SPEC), positive predictive value (PPV), negative predictive value (NPV), accuracy (ACC) and balanced accuracy (BACC). In addition, the significance of ML models incorporating preprocessing rules and models without rules was calculated by ANOVA test (Microsoft Office 365) which yielded dedicated p-values with significance threshold of *p* < 0.05, as well as the standard deviation, mean value and confidence interval (CI 95%) (Supplemental Table S3).

## Results

### Data preprocessing pipelines

The ML models built over the MLDP preprocessing approach for predicting the 24-months progression in DLBCL cancer cohort utilized only one preprocessing step in the setups with the “pref-remove” and “pref-keep” directives, as well as without included RST. The more complex pipelines with higher count of involved DP algorithms were recorded in the models with “exp-keep” (three) and “exp-remove” (four) directives. The results demonstrated the high complexity of data preprocessing pipelines (6–7 incorporated algorithms) for both 36-months survival ML predictive models in glioma cancer, and for predicting the low-high risk in prostate cancer patients. The feature selection (68% glioma, 74% prostate) and outlier detection (71% glioma, 66% prostate) algorithms demonstrated the highest average occurrence across all folds. The SMOTE (44% glioma, 37% prostate) and random undersampling (39% glioma, 40% prostate) methods demonstrated significant average occurrence in the preprocessing pipelines for both aforementioned cohorts. See Supplemental Table S4 for pipeline details of trained ML models.

### Feature analysis

#### 24-months progression in DLBCL cancer

The presence of domain-expert input features in MLDP-based ML models for 24-months progression in DLBCL prediction was observed, where all five features (TLG_g_Total, Coarseness, Busyness, HardArea_Volume, Max_Diameter_mm_VOI) were contained in the models without RST (feature count *n* = 28), as well as in models with “pref-keep” (*n* = 28) and “exp-keep” (*n* = 28) directives. The model with “pref-remove” (*n* = 5) instruction contained only the “Max_Diameter_mm_VOI”. The manually preprocessed data (manual + RST) was restricted to the 10 most prominent features for ML training. The models with “pref-keep” and “exp-keep” directives contained all input features, while the model without RST and with “pref-remove” directive included only two input features.

#### 36-months survival prediction of glioma cancer

For predicting the 36-months survival prediction in glioma cancer, all of the input features were present across the MLDP-based models with “pref-keep”, “pref-remove” and “exp-keep” directives and without RST. The manually preprocessed data was restricted to 20 features where ML models with “exp-keep” directive included all prominent features, while models without RST and “pref-keep” directive incorporated four and model with “pref-remove” instruction contained three prominent features.

#### High-vs-low risk prediction in prostate cancer

For predicting the high-vs-low risk in prostate cancer patients, all of the input features were present across the models without RST and with “pref-keep”, “pref-remove” and “exp-keep” directives in MLDP-based ML model training. The manually preprocessed data was restricted to 20 features, where all models except “exp-remove” instruction included all input features.

Overall, the input features showed no presence in any of the models with “exp-remove” directive as was expected. See Table 2 for detailed overview of prominent features enclosure in all trained predictive models in both manual and MLDP setup. See Supplemental Table S5 for detailed feature overview of models built with MLDP preprocessing and Supplemental Table S6 for detailed feature overview of models built with manual preprocessing.

**Table 2:**
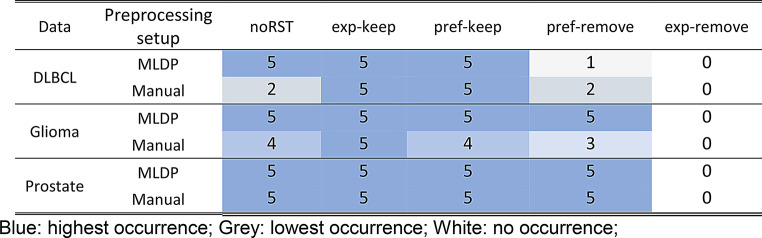
The occurrence of five most prominent features determined in retrospective studies of all three analyzed cohorts (glioma, prostate, DLBCL) and across all trained predictive models. MLDP – ML-driven data preparation; rule set table (RST); DLBCL – diffuse large B-cell lymphoma

### Performance evaluation

#### 24-months progression in DLBCL cancer

For predicting 24-months progression in DLBCL cancer patients, the highest performance across all model schemes was achieved by the MLDP + RST XGBoost model (81% BACC), compared to the 54% BACC (exp-remove), 78% BACC (pref-keep) and 65% BACC (pref-remove) models with the same setup (Table 3). Additionally, in manual + RST setup, The highest performance across all model schemes was achieved by the ML model with RST (pref-keep) (79% BACC), compared to 76% BACC (without RST), 76% BACC (exp-keep), 72% BACC (pref-remove) and 50% BACC (exp-remove). Overall, the ML models with MLDP + RST outperformed manual + RST models with “exp-keep” directive (81% BACC vs. 76% BACC), while underperformed with “pref-remove” directive 65% BACC (vs. 72% BACC) for DLBCL cohort.

**Table 3:**
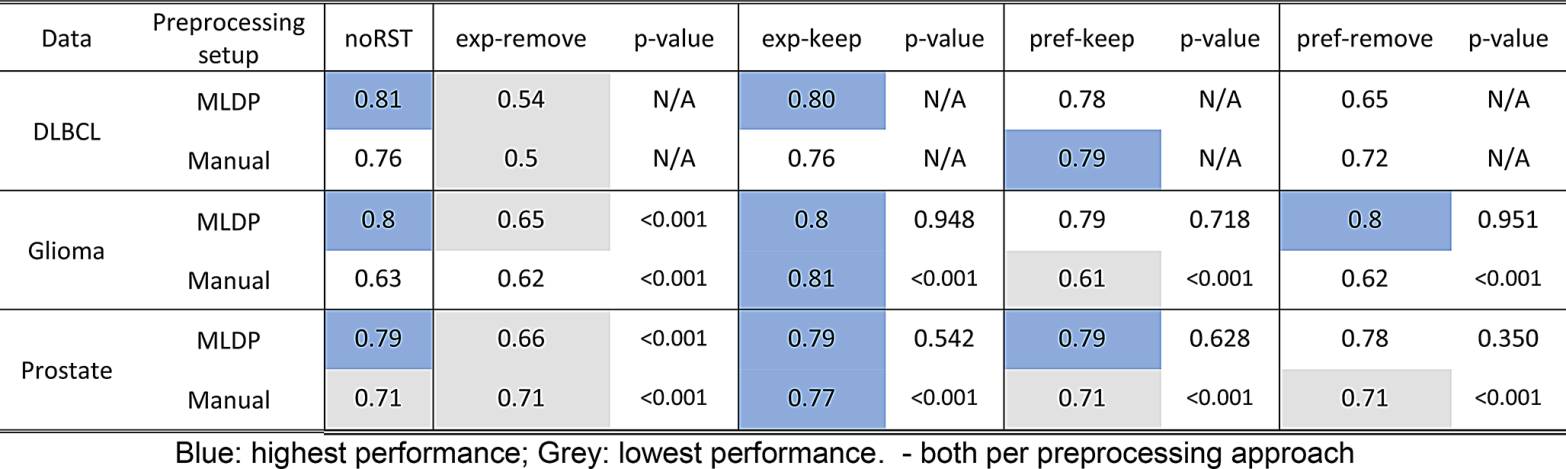
Predictive performance expressed in balanced accuracy (BACC) of all trained predictive models (XGBoost) with their p-values in both manual and ML-driven data Preparation (MLDP) schemes. Both schemes included models with (exp-keep, exp-remove, pref-keep, pref-remove) and without incorporated rule set table (RST). The “norst” scenario was the base of p-value predictive performance analysis tests, thus, it has no p-values. Furthermore, the DLBCL cohort being dual-centric, has no 100-fold cross-validation variants, thus, p-values would not be acquired. DLBCL – diffuse large B-cell lymphoma

#### 36-months survival prediction of glioma cancer

The model with the MLDP + RST (exp-keep) setup demonstrated the highest performance (80% BACC) for 36-months survival prediction in glioma cancer patients (Table 3). In the manual + RST setup, the ML model with “exp-keep” directive yielded highest performance (81% BACC, *p* < 0.001), while models with other directives ranged 61 − 63% BACC.

In the comparison of MLDP + RST and manual + RST setups, glioma models performed similarly with “exp-keep” (80% BACC vs. 81% BACC), while “pref-keep” and “pref-remove” yielded higher performance 79% and 80% BACC (vs. 61% and 62% BACC) respectively (Table 3).

#### High-vs-low risk prediction in prostate cancer

The model with the MLDP + RST (exp-keep) setup demonstrated the highest performance for predicting the high-vs-low risk in prostate patients (79% BACC). In manual + RST scheme the highest performance was achieved by ML models “exp-keep” directive (77% BACC, *p* < 0.001) (Table 3). Overall, prostate models with MLDP + RST outperformed manual + RST preprocessing across all directives, averagely 79% vs. 74% BACC. See Supplemental Table S7 for the performance metrics across all evaluated ML models.

ML models built with the XGBoost classifier demonstrated highest predictive performance across all cohorts (Table 3), compared to other classifiers (Supplemental Table S10).

## Discussion

In this study we proposed a data preprocessing approach which is capable of incorporating domain-expert directives in the decision-making procedures to improve the training ability of predictive ML models. The effects of such approach were investigated across two single and one dual-center datasets where we achieved the performance increase of ML models with manual preprocessing combined with RST of up-to 18% BACC compared to models without RST. The ML models trained with XGBoost algorithm outperformed models trained with other classical ML algorithms (RF, SVM) across all cohorts. Such results are in alignment with previous studies who performed ML analysis on these cohorts [12]. Overall, the ML models across all analyzed datasets benefited from RST, where glioma models (with “pref-keep”) demonstrated the highest performance increase (18% BACC), while DLBCL models benefited the least (3% BACC). Furthermore, the ML models with “exp-keep” (+ 18% BACC (glioma), + 6% BACC (prostate)) and “pref-keep” (+ 3% BACC (DLBCL)) instructions showed higher performance benefit compared to other models across all datasets. Given that the subsets of prominent features for all involved cohorts were already determined in previous studies [15–17], resulted in training the high-performing ML models, such fact was used as a strong reference point to test the RST method by intentionally including these features to obtain an independent confirmation that the RST is indeed functional. Otherwise, it would be difficult to impossible to measure the results observed from the RST, and consequently if the RST approach functions as intended. Thus, we argue that the aforementioned results validate the general functioning of the RST approach.

Furthermore, these findings confirm the hypothesis of our study, namely, that clinician involvement in the DP steps through RST is not only feasible, but necessary if one incorporates manual data preprocessing steps. On the other hand, the MLDP with RST models have not demonstrated an added value compared to the models built solely on the MLDP approaches without RST. Such scenario occurred due to the fact that MLDP performs both ML-driven data preprocessing and hyperparameter optimization, hence the most favorable solution is achieved automatically. Nevertheless, this high-performance of MLDP comes with a cost of highly-complex DP processing chains, that make them less-interpretable and repeatable. In contrast, the importance of RST appears to be magnified if clinical experts wish to guide the data preprocessing step with explicit rule sets according to standard clinical knowledge or latest scientific consensus and with relying on a manual DP pipeline for assuming more control over the whole data preprocessing step. Irrespective of the above, MLDP + RST-guided models may serve as validation tools for different clinical hypothesis, where clinicians can test the importance of features with explicit rule set instructions (e.g. exp-keep vs. exp-remove).

Even though the manual preprocessing steps contained fixed pipelines with incorporated algorithms for outlier detection, feature selection and class imbalance correction as the most commonly utilized approaches in similar works, the ML models built on such data underperformed across all datasets and action directives compared to MLDP. Nevertheless, including the RST directives in manual preprocessing setup, increased the ML performance by 3 − 18% BACC across all cohorts, implying that RST input does have a positive effect on ML training regardless of the preprocessing scheme. Based on this observation we can verify that performance based on MLDP + RST does not arise from the general ability of the MLDP system to optimize pipelines and hyperparameters of employed algorithms, but it clearly confirms the added value of RST input.

We observed that preprocessing pipelines for MLDP + RST model training contained on average five incorporated algorithms, therefore such phenomenon confirms the premise that data preprocessing is indeed a complex task which cannot be easily standardized and shall be considered as a non-deterministic approach which requires in-depth expert knowledge.

In order to obtain better understanding of the effects of incorporated rules, the feature occurrence analysis was conducted. The results revealed that for glioma cancer prediction, the ML model that benefited the most from RST (exp-keep) combined with manual preprocessing, represented the single case which incorporated all five input features (*n* = 5) compared to other scenarios (range of *n* = 3–4). This model contained an explicit instruction “exp-keep”, which prevented the ML algorithms to alter the feature subset in a way that the features determined by clinical expert as essential are removed. Consequently, the aforementioned model demonstrated the highest predictive performance (81% BACC) in a cross-validation environment.

Even though the models with incorporating RST (with pref-keep/exp-keep directives) demonstrated similar or increased performance compared to the ones without RST across all cohorts, the models built on the “pref-remove” directives in glioma and DLBCL underperformed in comparison to the no RST models (62% BACC vs. 63% BACC (manual preprocessing glioma), 72% BACC vs. 76% BACC (manual preprocessing DLBCL) and 65% BACC vs. 81% BACC (MLDP preprocessing DLBCL). This implies that external inputs may have high implications on the ML algorithm decisions, and may cause negative effects on the ML training processes when they are suboptimal. Even though the “pref-remove” directive is not an explicit instruction but rather a clinician’s preference, the feature selection algorithm’s decision is based on the internal criteria, hence if the feature of interest is ranked below the defined threshold (Supplemental Table S1), it will be removed.

To date, multiple studies investigated the incorporation of domain knowledge into the ML model building process across various non-medical fields [27–32], while only a very few studies explored such knowledge input within the medical domain, mostly in electronic health record model generation, structural health monitoring or text-to-image generation based on large language models [33–36]. Similar works concentrating on medical image analysis incorporated domain knowledge in AI analysis approaches. Various studies utilized convolutional neural networks (CNNs) as feature extractors from natural datasets, where features are further provided into a linear classifier which is trained over medical imaging datasets [37–40]. The main applications of such transfer-learning approach were found in mammography lesion classification, skin cancer differentiation and the identification of chest pathology [41–43]. Guan et al. investigated the incorporation of the three-staged pattern into the neural network, which is based on the radiological procedure of how clinicians read medical images [44]. Li et al. investigated the “attention maps” approach, which is based on annotating the focus areas of medical doctors during the diagnosis establishment from medical images [45]. This approach was used in glaucoma diagnosis where “attention map” was incorporated into the AG-CNN, and the results demonstrated significant performance increase of 0.09 AUC in comparison to the current state-of-the-art methods. Similarly, Fang et al. reported the performance gain of 8% accuracy of the lesion awareness classification in the optical coherence tomography (OCT) images [46]. Hussein et al. suggested six most prominent “hand-crafted” features with tight malignancy score connection (calcification, sphericity, margin, lobulation, spiculation and texture) to be included into the ML classification of the malignant-benign risk assessment in lung nodules [47]. Similarly, the hand-crafted features were combined with the CNN-based extracted features to train the ML algorithm for lung nodule classification in the study proposed by Xie et al., where authors reported the performance increase of roughly 4% accuracy compared to the model without hand-crafted features included [48].

In contrast to the above-mentioned studies, our study diverges in the following ways: First, it enables direct influence of the clinical expert on the algorithms’ data preprocessing decision-making phases both with direct or preferential (non-explicit) rules. Second, by utilizing non-explicit rules, it allows a direct clinician vs. AI preferential analysis where clinical domain relevance can be combined with mathematical importance, albeit, keeping the process controlled by the clinician. Third, this approach also provides the possibility for hypothesis testing, where clinician preferences or hypotheses about what features may be relevant for characterizing the disease can be directly tested against AI decision-making processes. As an example, for DLBCL cancer cohort, the primary largest lesion was selected per patient. However, the RST would be a perfect tool to test the primary vs. multiple lesions hypothesis, where a clinician could use e.g. pref-keep directive to prefer multi-lesion radiomic features against primary ones, and the ML itself would remain a decision-maker which would provide the answer on which approach (multi-lesion or primary-lesion radiomics) would yield best performance. Other example would be the case when there is a suspicion that a certain feature X is clinically more relevant then feature Y, then the clinician can express hes/her preference to keep the certain feature, even if it is not the highest ranking within its redundant feature group.

The main limitation of our study is in its retrospective nature, where the highest-ranking features were already known, without including the actual clinicians’ input, however, this knowledge was essential to have a ground truth reference to be able to properly validate the proposed method. Additionally, while some cohorts were single-center, we wish to emphasize the aim of the study, namely, to investigate the added value of RST and not to propose clinically-utilizable prediction models.

## Conclusion

The proposed study demonstrated the added value of our RST concept to classification performance increase by incorporating clinical domain knowledge into the data preprocessing decision-making process. The inclusion of such information, if present, should be considered as important part of the data preprocessing pipeline in future clinical ML analysis scenarios. Relying in the RST concept allows clinicians to gain control over the data preprocessing phase of AI studies, while keeping the given pipeline as simple as possible.

## Electronic supplementary material

Below is the link to the electronic supplementary material.

Supplementary Material 1

## Data Availability

Contact the corresponding author for data access-related queries, or refer to [12].

## Code availability

From the corresponding author upon reasonable request. The software for testing purposes can be accessed at: https://github.com/dKrajnc/RST.
