# Supplementary material for: Clinician-driven automated data preprocessing in nuclear medicine AI environments
Source: Eur J Nucl Med Mol Imaging. 2025 Mar 7;52(9):3444–54. doi: 10.1007/s00259-025-07183-5 (PMC12222405; doi:10.1007/s00259-025-07183-5)
Supplement: Supplementary file 1 — Supplementary Material 1 [file 259_2025_7183_MOESM1_ESM.docx]

Clinician-driven automated data preprocessing in nuclear medicine AI environments

SUPPLEMENTAL

Authors: Denis Krajnc^1^, Clemens P. Spielvogel^2^, Boglarka Ecsedi^3,1^, Zsombor Ritter^4^, H. Alizadeh^5^, Marcus Hacker^2^, Laszlo Papp^1, *^

^1^Center for Medical Physics and Biomedical Engineering, Medical University of Vienna, Vienna, Austria

^2^Department of Biomedical Imaging and Image-guided Therapy, Division of Nuclear Medicine, Medical University of Vienna, Vienna, Austria

^3^Georgia Institute of Technology, Atlanta, GA, USA

^4^Department of Medical Imaging, University of Pécs, Medical School, Pécs, Hungary

^5^University of Pécs, Medical School, 1st Department of Internal Medicine, Pécs, Hungary

Correspondence:

*Laszlo Papp, PhD

Applied Quantum Computing Group, Center for Medical Physics and Biomedical Engineering, Medical University of Vienna, Vienna, Austria

Währinger Gürtel 18- 20, 1090 Vienna

laszlo.papp@meduniwien.ac.at

**Supplemental S1: The decision-making criteria designed specifically for each preprocessing algorithm**

The decision-making criteria designed specifically for each preprocessing algorithm described for both sample (SAMPLE-X) and feature (FEATURE-Y) space directives. The criteria are considered for non-decisive rule set table (RST) directives such as include and exclude respectively. For the sake of simplicity, KEEP and REMOVE rules are not shown here, as they are explicit rules.

|  | Sample space | | Feature space | |  |
| --- | --- | --- | --- | --- | --- |
| Method | pref-keep | pref-remove | pref-keep | pref-remove | NOTE |
| OD | SAMPLE-X | | FEATURE-Y | | pref-keep/pref-remove SAMPLE-X: choose/reject SAMPLE-X if in a range of pre-defined threshold compared to the fixed threshold (0.6 suggested by the literature) against similarly-ranked samples.  pref-keep/pref-remove FEATURE-Y: Choose/reject FEATURE-X if in a range of pre-defined threshold of the Isolation Forest algorithm against other similar features. |
| DR | N/A | | FEATURE-Y | | pref-keep/pref-remove FEATURE-Y: Is feature in a range of pre-defined threshold or magnitude of a fixed threshold? (e.g., preservation percentage of 95%) within the PCA algorithm? The principal component including FEATURE-Y needs to be preferred against other similar principal components to make the inclusion/exclusion. |
| US | SAMPLE-X | | N/A | | pref-keep/pref-remove SAMPLE-X: prefer inclusion/exclusion against other samples within the Tomek Links approach if similarly ranked to those samples. |
| OS | SAMPLE-X | | N/A | | pref-keep/pref-remove SAMPLE-X: include/exclude the sample unless KNN distance is higher than average distance. |
| FS | N/A | | FEATURE-Y | | pref-keep/pref-remove FEATURE-Y: Is feature in a range of a pre-defined threshold or magnitude of a fixed threshold? If yes, choose/reject FEATURE-Y against other similar features. |

OD – Outlier detection; DR – Dimensionality reduction; US - Undersampling; OS - Oversampling; FS – Feature selection; kNN – K nearest neighbour;

**Supplemental S2: Hyperparameters of utilized data preparation algorithms**

List of hyperparameters for data preparation algorithms. Auto parameter with value set to true allows full scale oversampling/undersampling utilization, achieving the 0% imbalance ratio. Only when set to false, oversampling technique may be instructed to up-sample the dataset by a certain percentage through the “oversampling percentage” parameter, or undersampling technique may be instructed to down-sample the dataset by a certain number of samples through the “undersampling amount” parameter.

| Algorithm | Hyperparameters | Value |
| --- | --- | --- |
| Outlier detection | Tree count *(input number)* | 1000 |
| Feature selection | Feature count *(input number)*  Rank method *(R-squared)* | UDI |
|  |  | RSquared |
| Oversampling | Neighbors count *(input number)*  m_neighbors count *(input number)*  n_neighbors count *(input number)*  Auto *(true/false)*  Oversampling percentage *(if auto = false then input number)*  Type *(Random Oversampling, SMOTE, BSMOTE)* | 3 |
|  |  | 5 |
|  |  | 7 |
|  |  | False |
|  |  | 100 |
|  |  | SMOTE |
| Undersampling | Type *(Random Undersampling, Tomek Links)*  Auto *(true/false)*  Undersampling amount *(if auto = false then input number)* | TomekLinks |
|  |  | false |
|  |  | UDI |
| PCA | Preservation percentage *(input number)* | 95 |

SMOTE – Synthetic minority oversampling technique; BSMOTE – Borderline synthetic minority oversampling technique; PCA – Principal component analysis; UDI – User-defined input;

**Supplemental S3: Statistical analysis of ML predictive models with and without data preparation**

Conventional statistical analysis of established ML predictive models with and without machine learning data preparation (MLDP) scenarios. For each scenario, models were train with (keep, remove, include, exclude) and without Rule Set Table (RST).

|  |  | Glioma | | Prostate | | DLBCL | |
| --- | --- | --- | --- | --- | --- | --- | --- |
| Model | Statistical method | Manual | With MLDP | Manual | With MLDP | Manual | With MLDP |
| noRST | mean ± σ | 0.80±0.01 | 0.80±0.01 | 0.77±0 | 0.79±0 | 0.77±0 | 0.82±0 |
|  | CI (95%) | 0.77 – 0.83 | 0.77 - 0.83 | 0.77 - 0.77 | 0.79 - 0.79 | 0.77 - 0.77 | 0.82 - 0.82 |
| Keep | mean ± σ | 0.62±0.02 | 0.80±0.01 | 0.72±0 | 0.79±0 | 0.77±0 | 0.82±0 |
|  | CI (95%) | 0.59 – 0.65 | 0.77 - 0.83 | 0.71 - 0.73 | 0.79 - 0.79 | 0.77 - 0.77 | 0.82 - 0.82 |
| Remove | mean ± σ | 0.62±0.02 | 0.65±0.02 | 0.71±0 | 0.67±0 | 0.68±0 | 0.52±0 |
|  | CI (95%) | 0.59 – 0.65 | 0.62 - 0.68 | 0.70 - 0.72 | 0.66 - 0.68 | 0.68 - 0.68 | 0.52 - 0.52 |
| Include | mean ± σ | 0.61±0.02 | 0.79±0.02 | 0.71±0 | 0.79±0 | 0.80±0 | 0.77±0 |
|  | CI (95%) | 0.58±0.64 | 0.76 - 0.82 | 0.70 - 0.72 | 0.79 - 0.79 | 0.80 - 0.80 | 0.77 - 0.77 |
| Exclude | mean ± σ | 0.62±0.02 | 0.80±0.01 | 0.72±0 | 0.78±0 | 0.73±0 | 0.73±0 |
|  | CI (95%) | 0.59 – 0.65 | 0.77 - 0.83 | 0.71 - 0.73 | 0.78 - 0.78 | 0.73 - 0.73 | 0.73 - 0.73 |

RF – Random Forest; MG – Multi gaussian; XGBoost – Extreme gradient boosting; NN – Neural networks; SVM – support vector machine; σ – Standard deviation; CI – Confidence interval;

**Supplemental S4: Data preprocessing pipelines of models built with MLDP**

S4.1: Data preprocessing pipelines of models built with ML-driven data preprocessing (MLDP) across all analyzed cohorts.

| Data | Setup | BSMOTE | FS | IF | RO | RU | SMOTE | TL | Count (%) |
| --- | --- | --- | --- | --- | --- | --- | --- | --- | --- |
| DLBCL | noRST | 0 | 0 | 0 | 0 | 0 | 0 | 1 | 0.14 |
|  | exclude | 0 | 1 | 0 | 0 | 0 | 0 | 0 | 0.14 |
|  | include | 0 | 0 | 0 | 1 | 0 | 0 | 0 | 0.14 |
|  | keep | 0 | 1 | 1 | 1 | 0 | 0 | 0 | 0.43 |
|  | remove | 0 | 1 | 1 | 0 | 1 | 1 | 0 | 0.57 |
| Glioma | noRST | 1 | 68 | 70 | 2 | 41 | 42 | 9 | 1.0 |
|  | exclude | 1 | 70 | 72 | 3 | 39 | 37 | 13 | 1.0 |
|  | include | 1 | 65 | 69 | 4 | 37 | 40 | 12 | 1.0 |
|  | keep | 1 | 67 | 73 | 5 | 39 | 44 | 6 | 1.0 |
|  | remove | 0 | 68 | 71 | 1 | 37 | 57 | 3 | 0.86 |
| Prostate | noRST | 1 | 79 | 64 | 4 | 40 | 40 | 6 | 1.0 |
|  | exclude | 1 | 73 | 68 | 8 | 38 | 39 | 6 | 1.0 |
|  | include | 1 | 71 | 63 | 2 | 48 | 36 | 5 | 1.0 |
|  | keep | 1 | 75 | 72 | 5 | 35 | 47 | 5 | 1.0 |
|  | remove | 3 | 70 | 65 | 4 | 41 | 25 | 13 | 1.0 |

SMOTE – Synthetic minority oversampling technique; BSMOTE – Borderline synthetic minority oversampling technique; PCA – Principal component analysis; TL – Tomek Links; RO – Random Oversampling; RU – Random Undersampling; IF – Isolation Forest; FS – Feature Selection; RST – Rule Set Table; DLBCL – diffuse large B-cell lymphoma;

S4.2 Average method occurrence across all data preprocessing pipelines in DLBCL models

S4.3 Average method occurrence across all data preprocessing pipelines in DLBCL models

S4.4 Average method occurrence across all data preprocessing pipelines in DLBCL models

**Supplemental S5: Feature occurrences of models with MLDP preprocessing**

Detailed overview of prominent features presence in all models build with ML-driven data preprocessing (MLDP) with (keep, remove, include, exclude) and without incorporated Rule Set Table (RST).


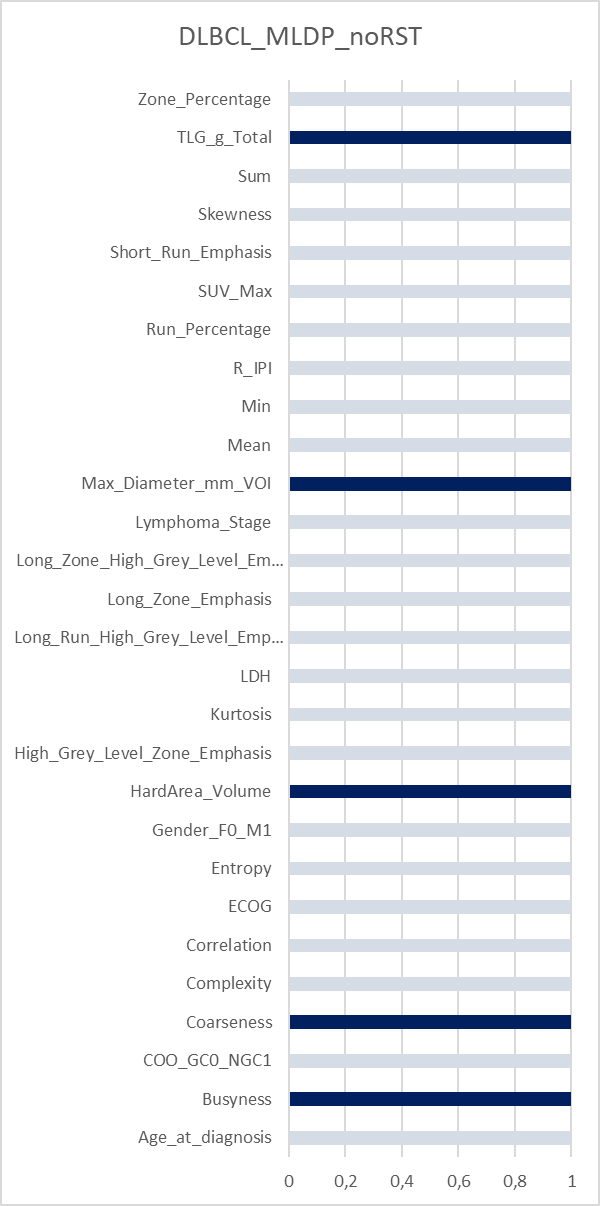

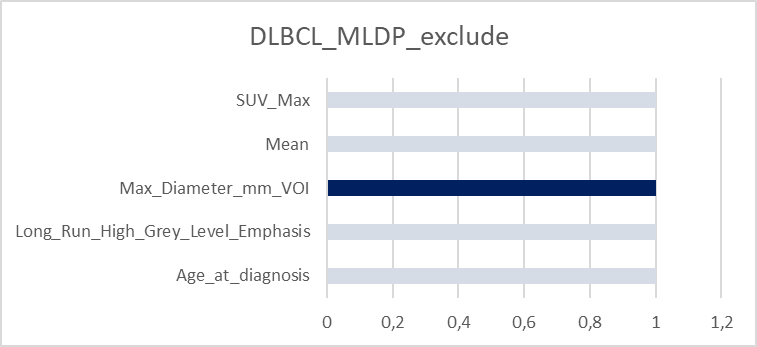


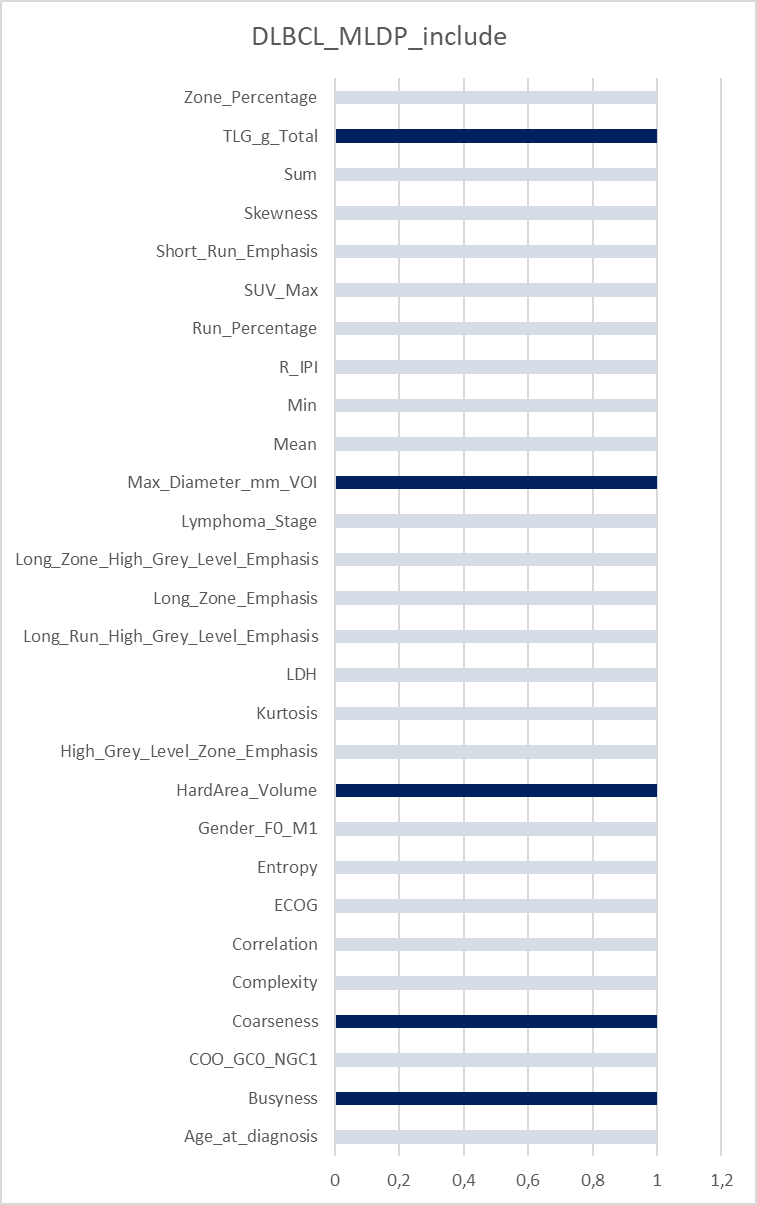

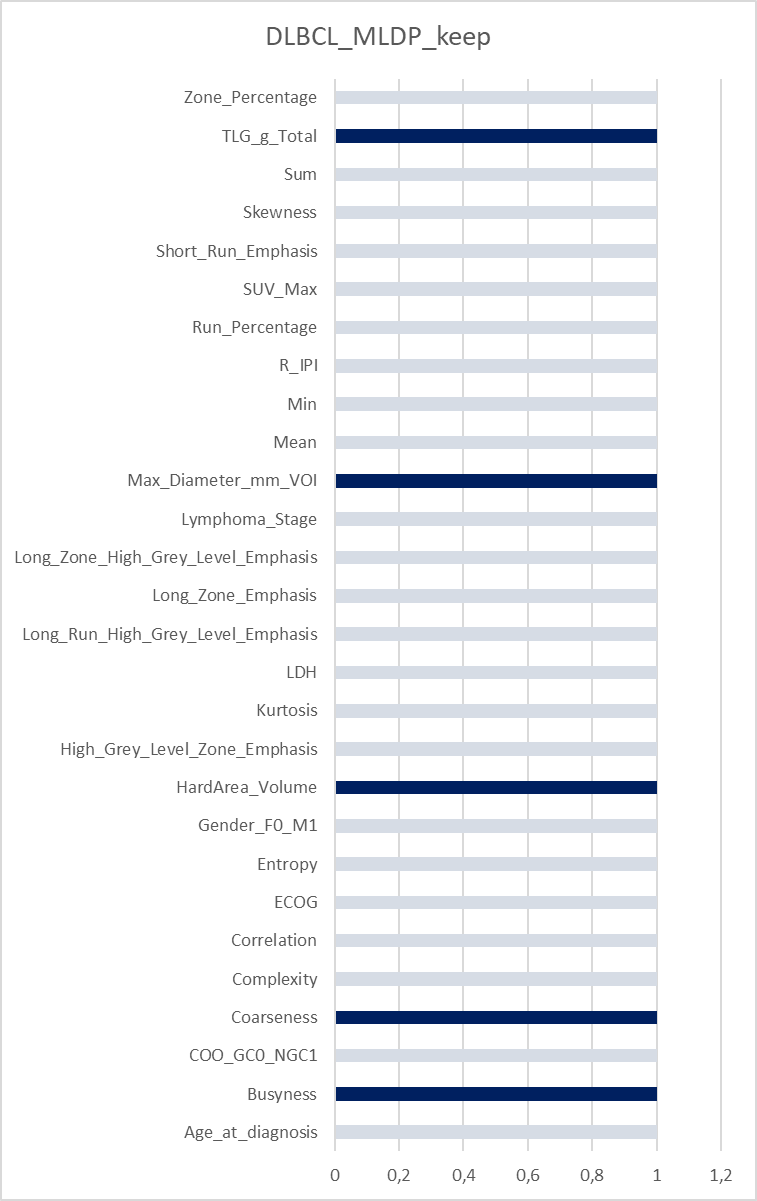


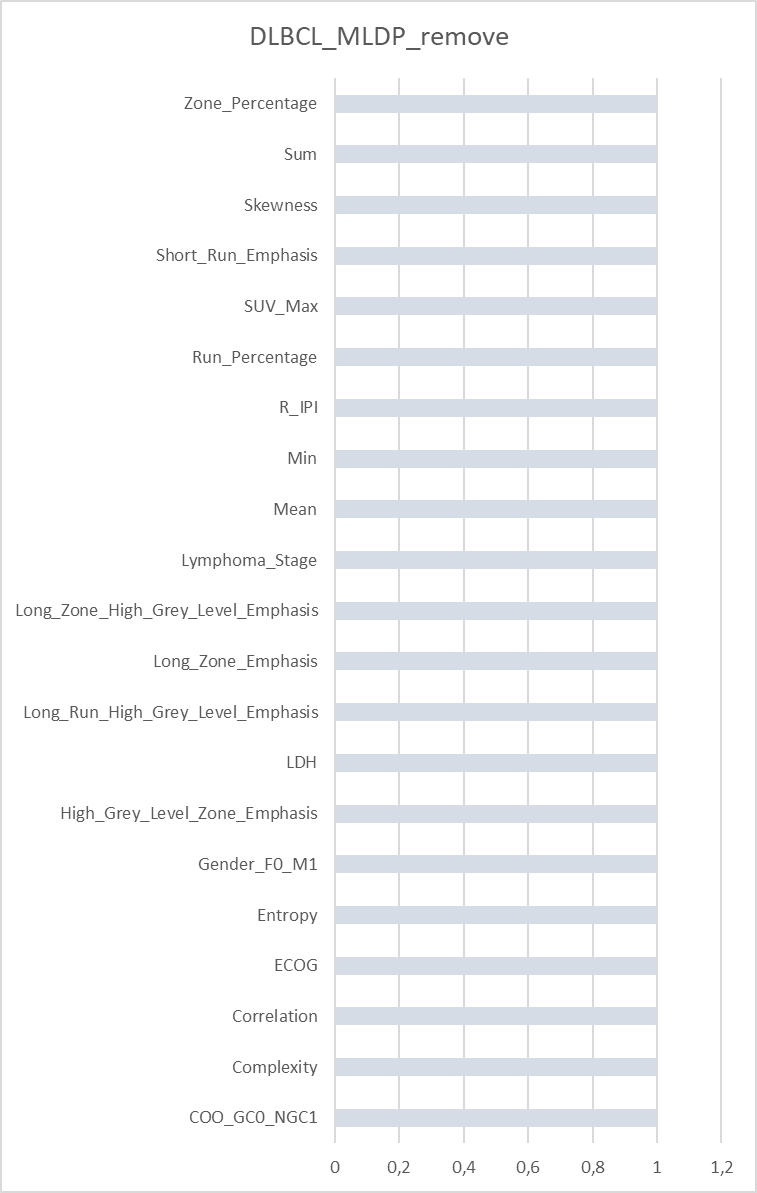


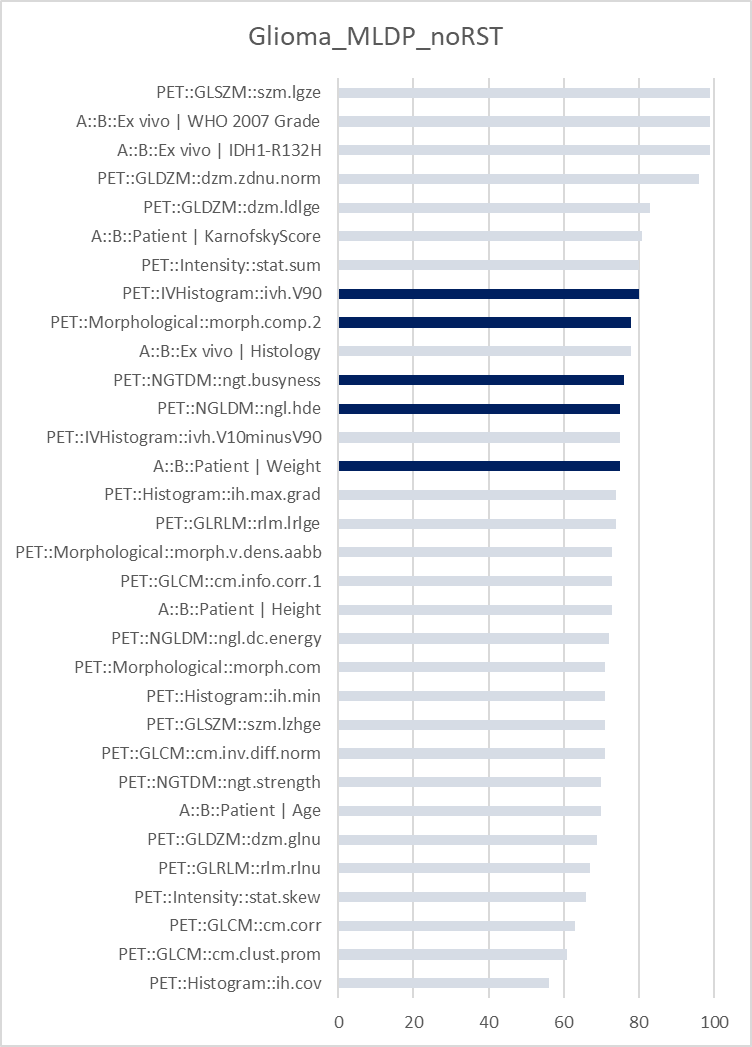

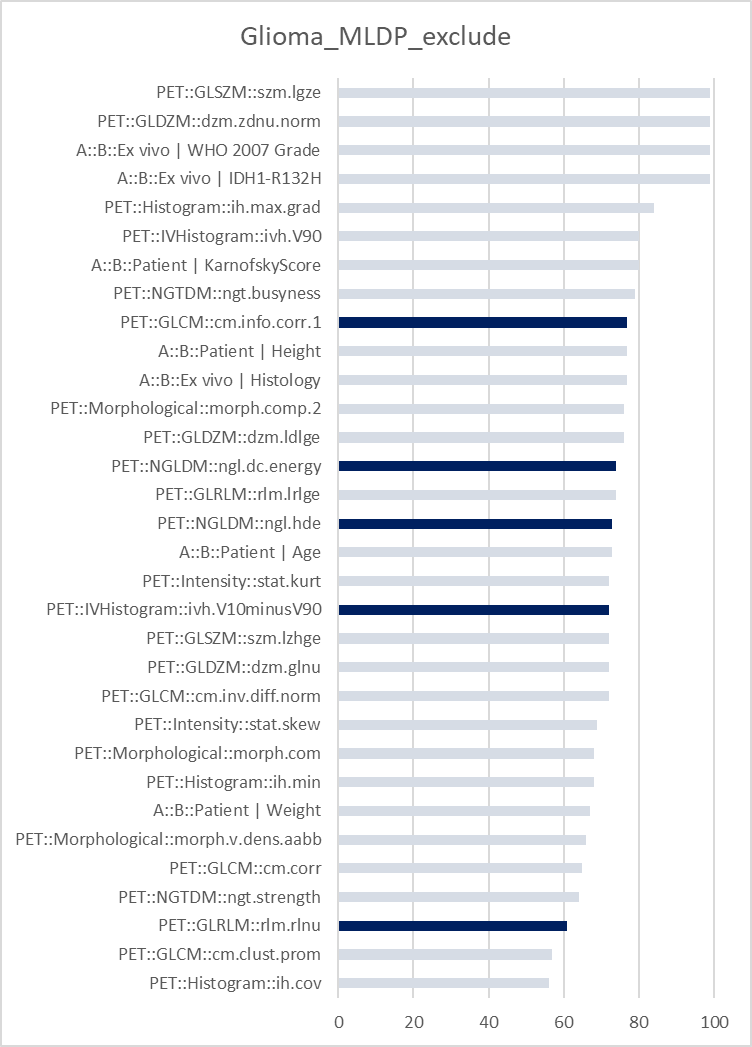


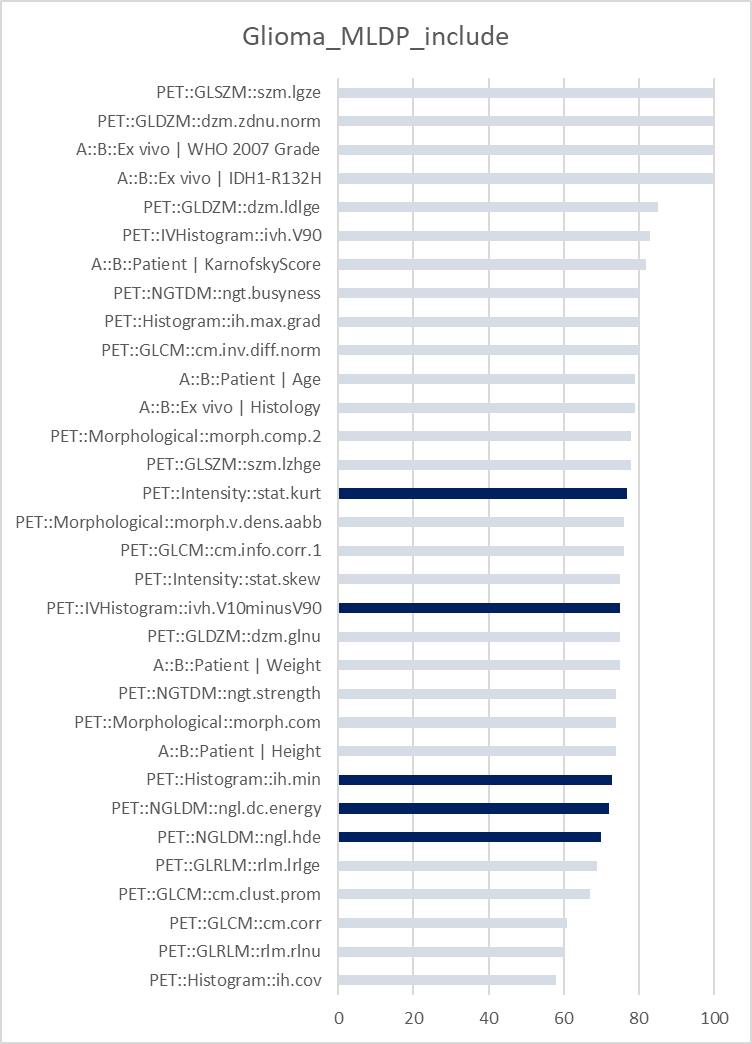

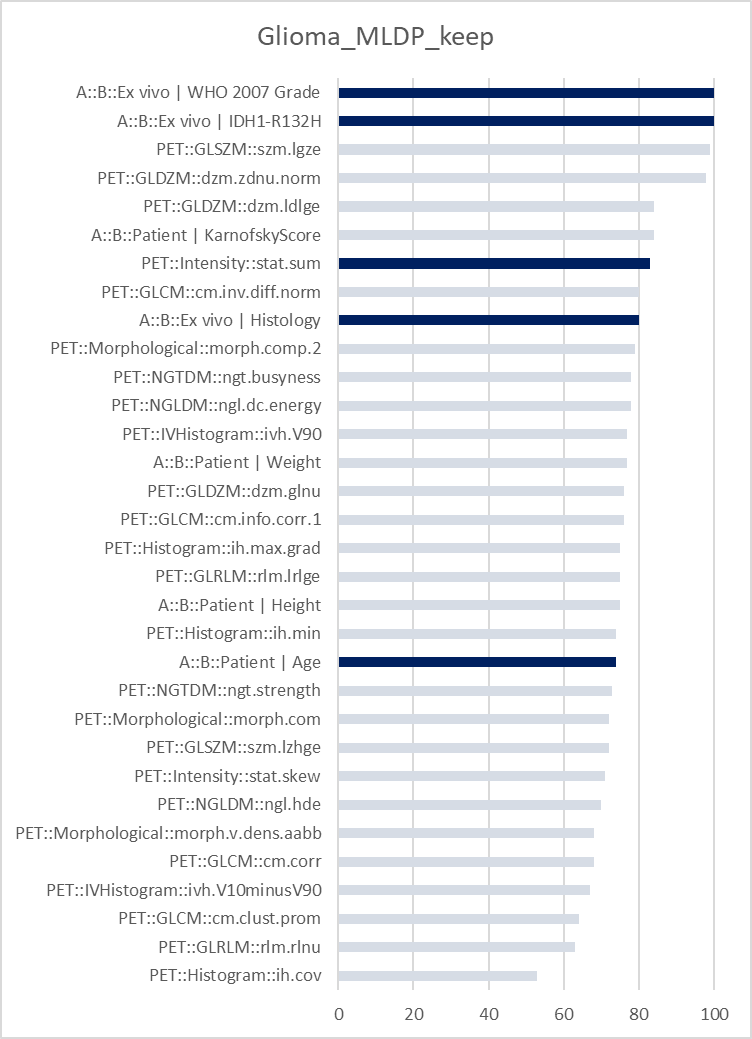


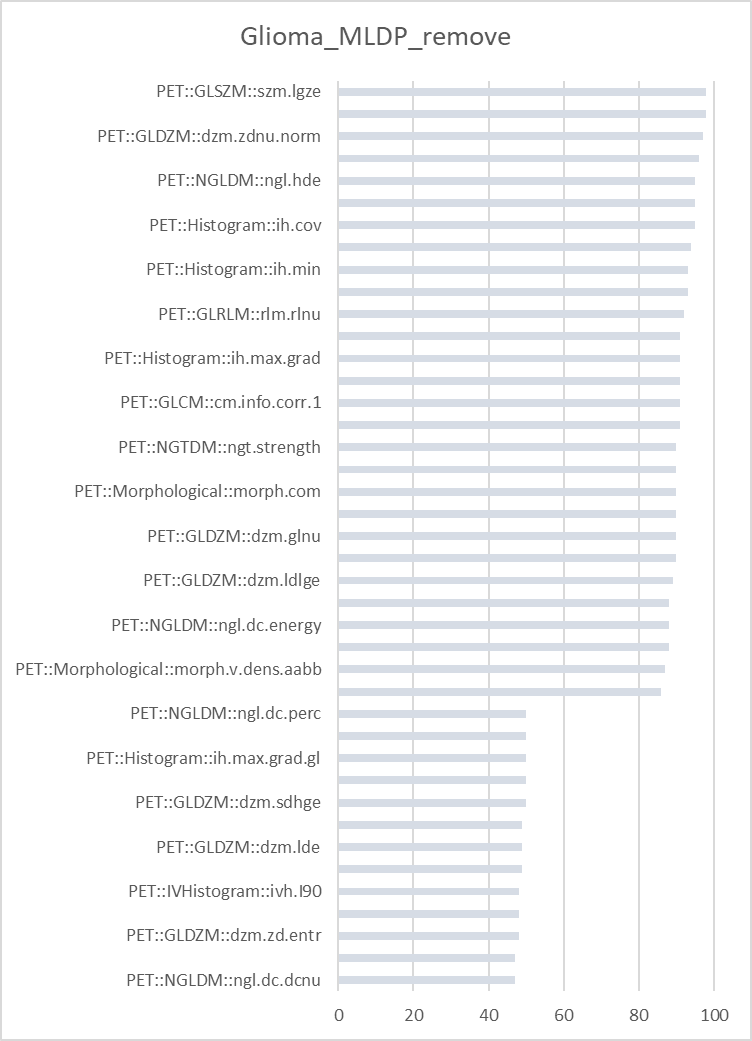


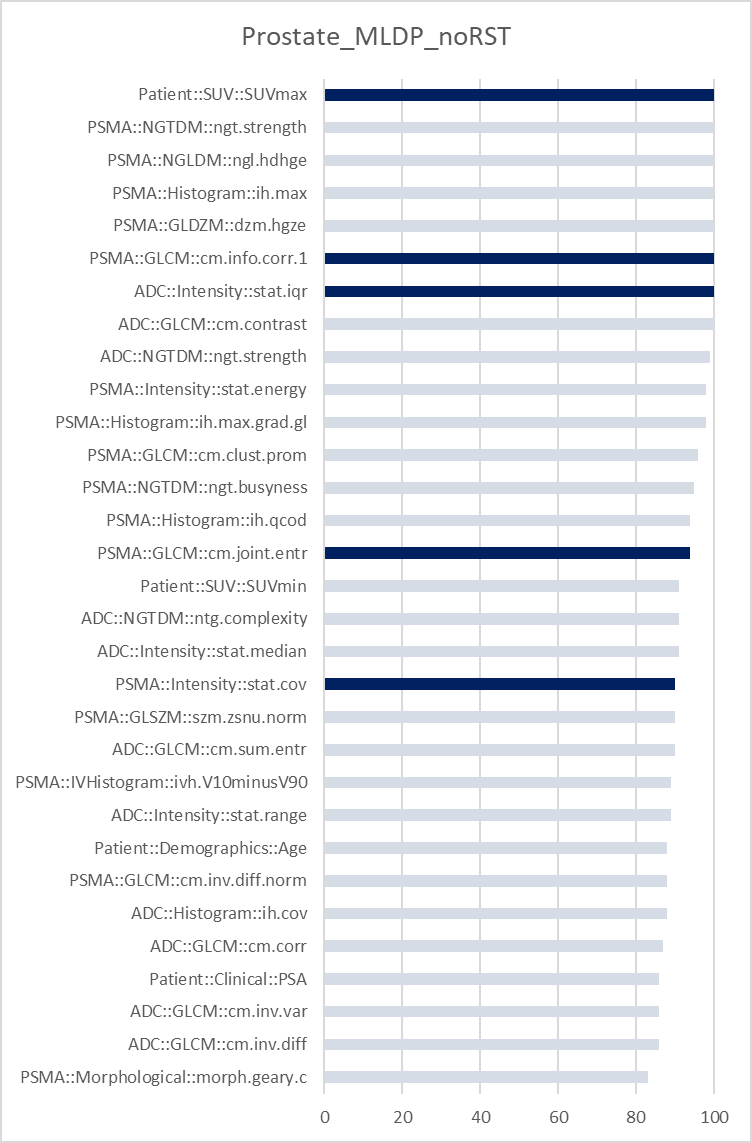

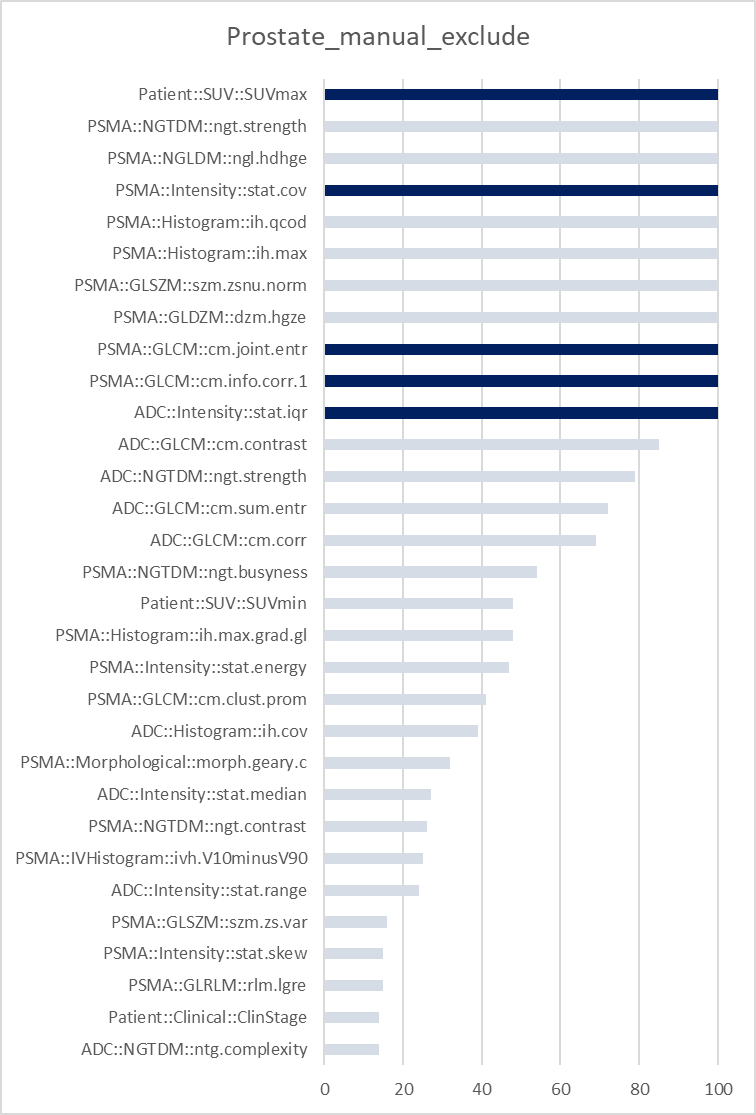


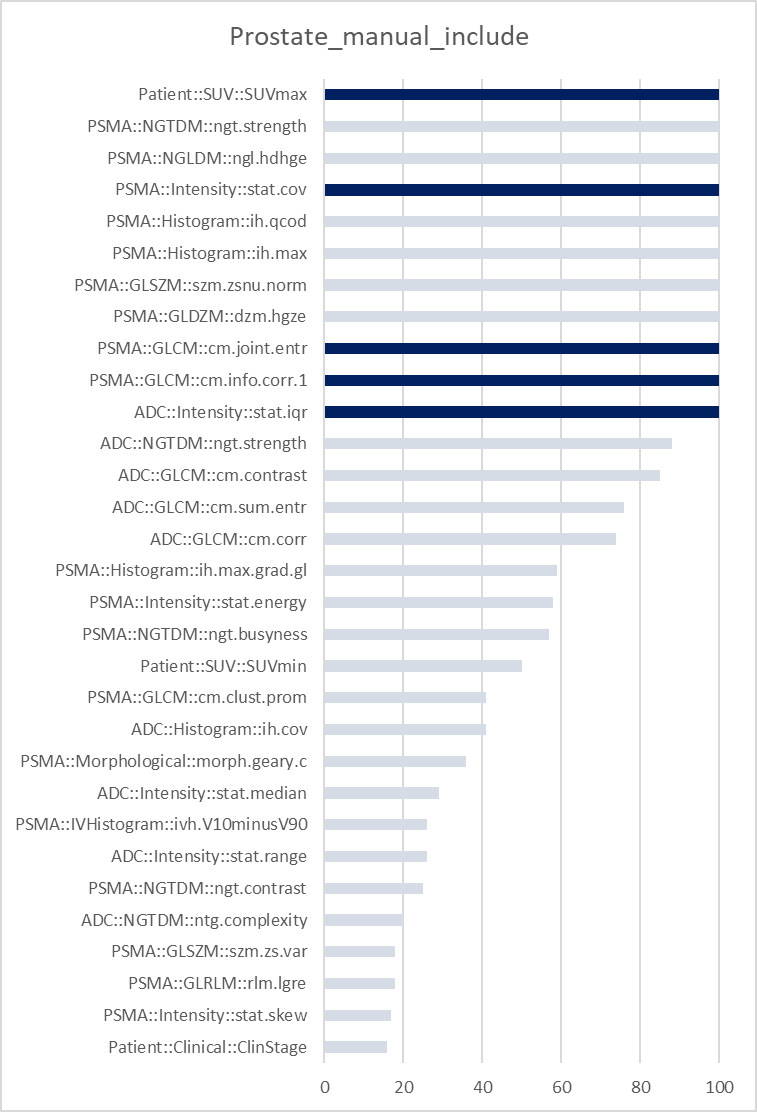

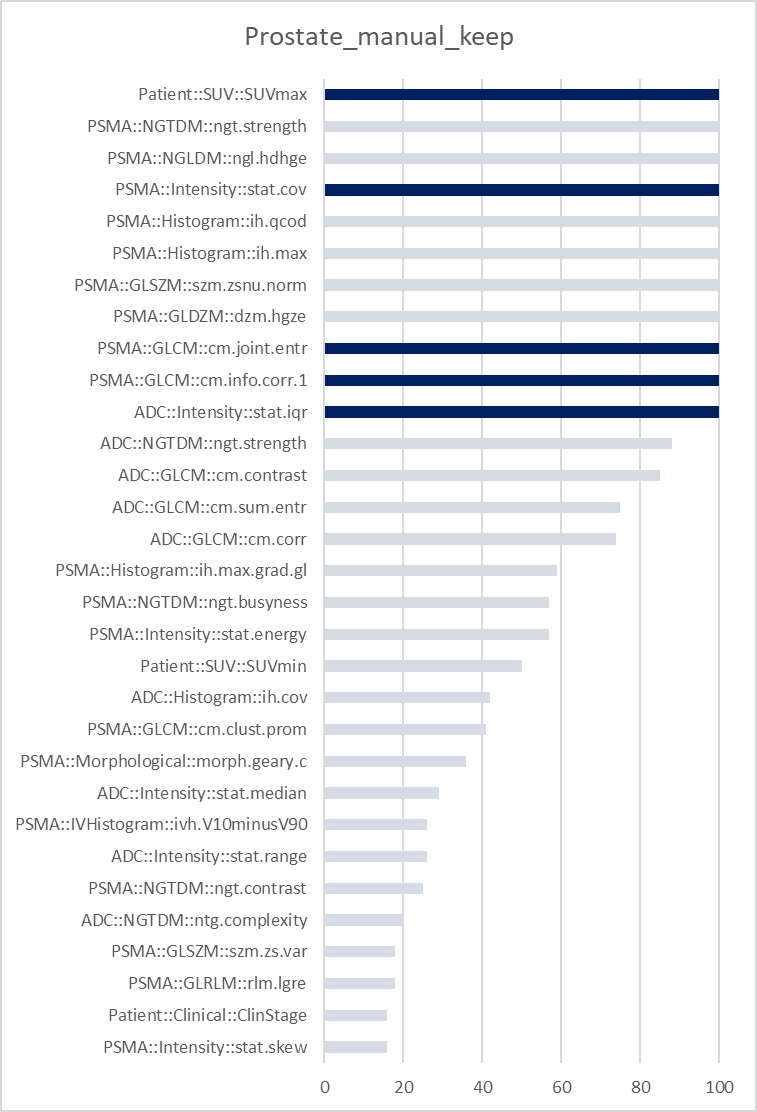


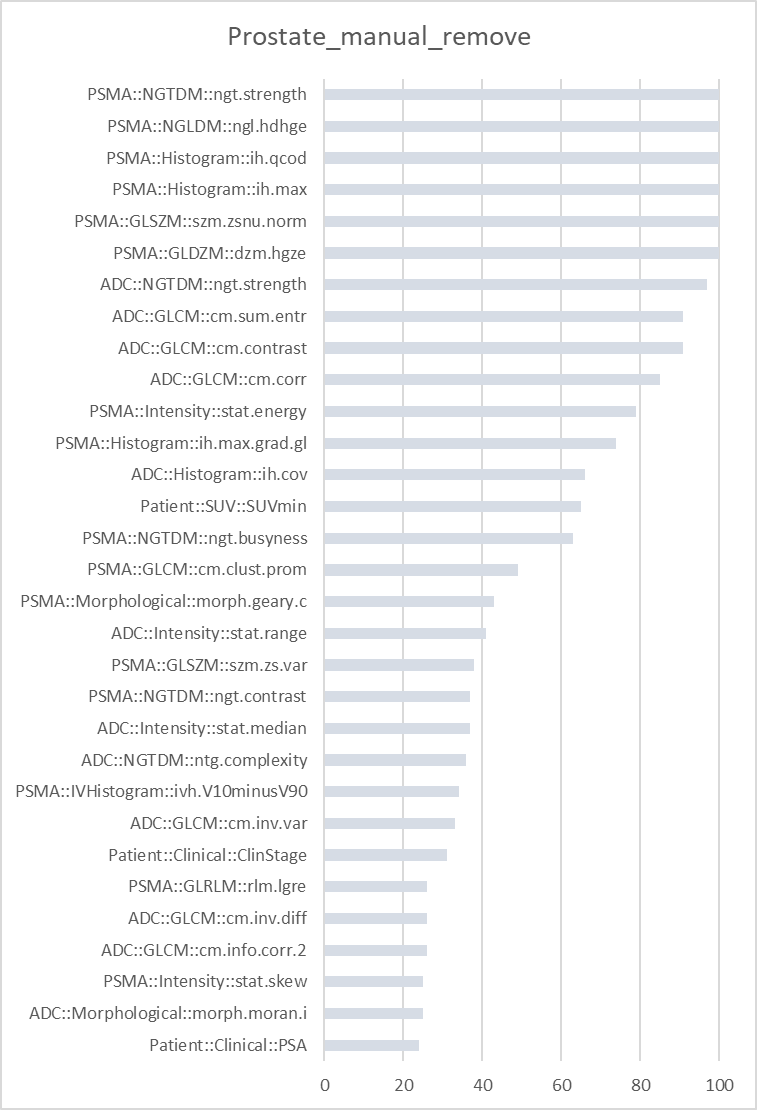


**Supplemental S6: Feature occurrences of models with manual preprocessing**

Detailed overview of prominent features presence in all models build with manual data preprocessing with (keep, remove, include, exclude) and without incorporated Rule Set Table (RST).


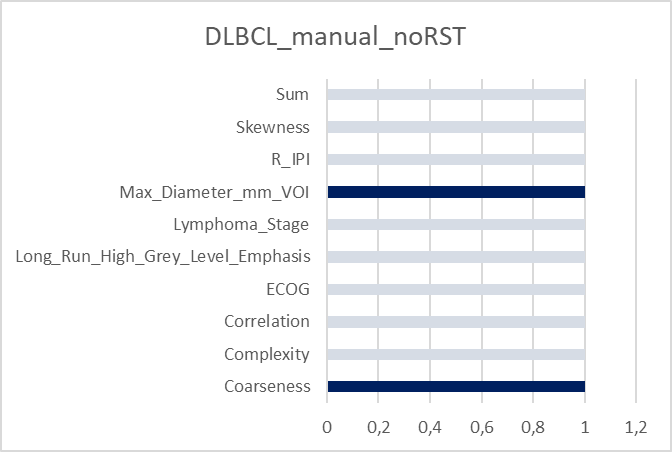

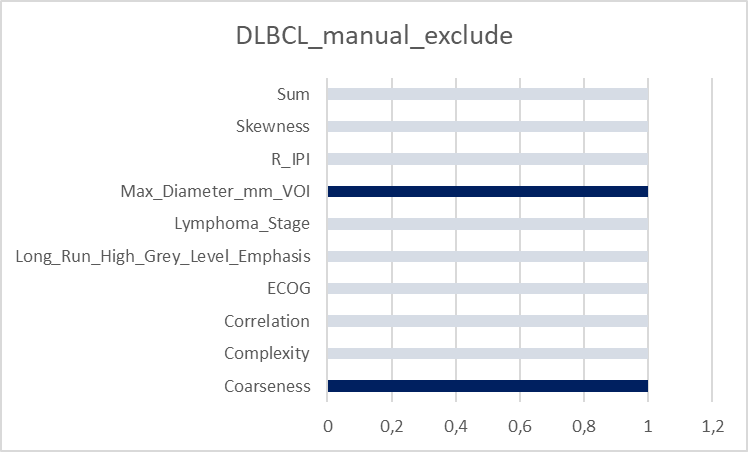


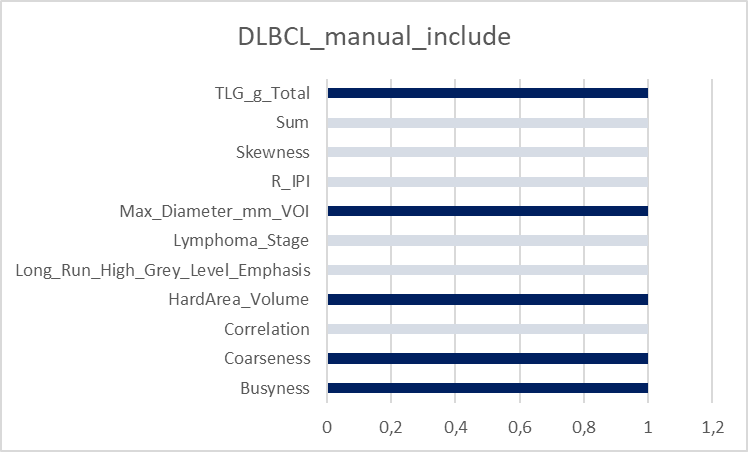

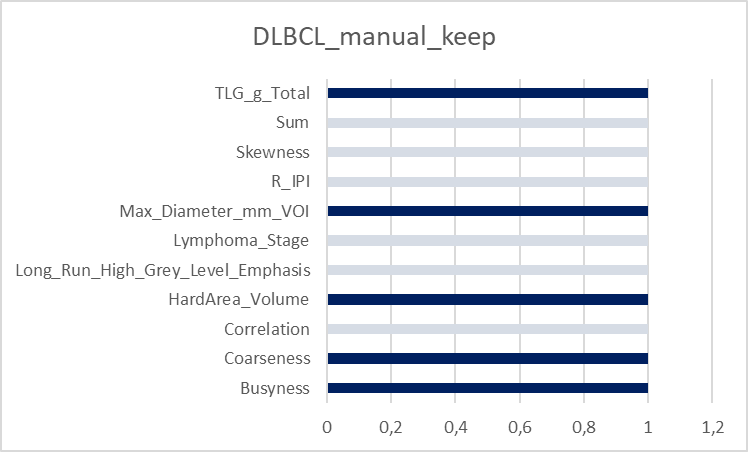


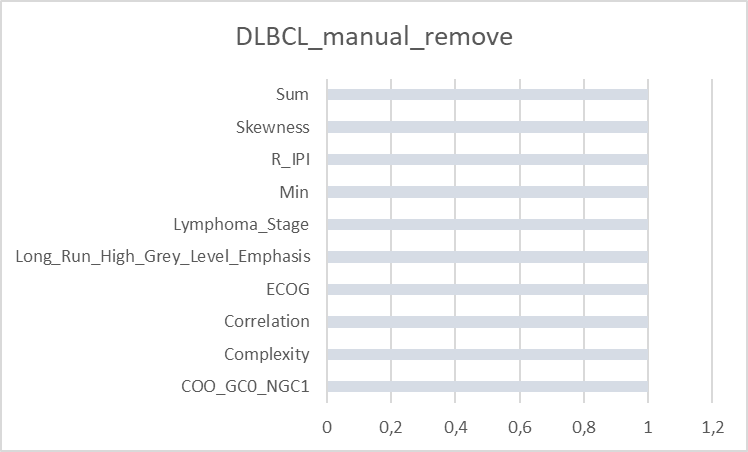


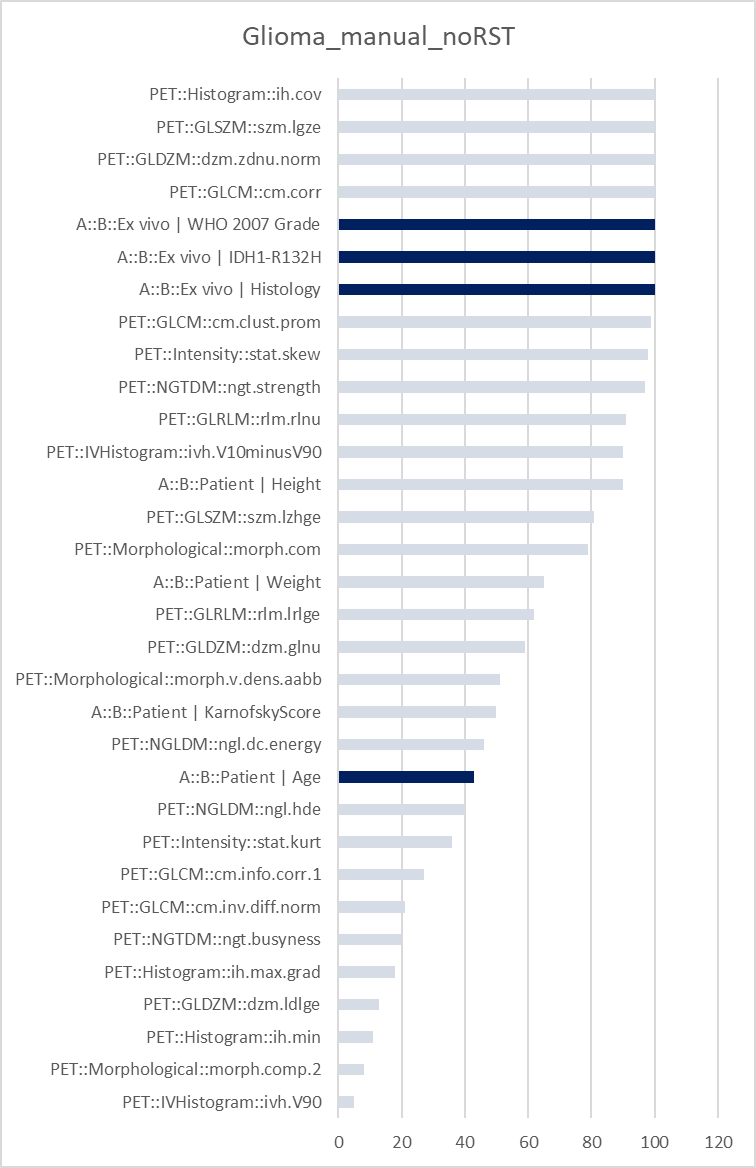

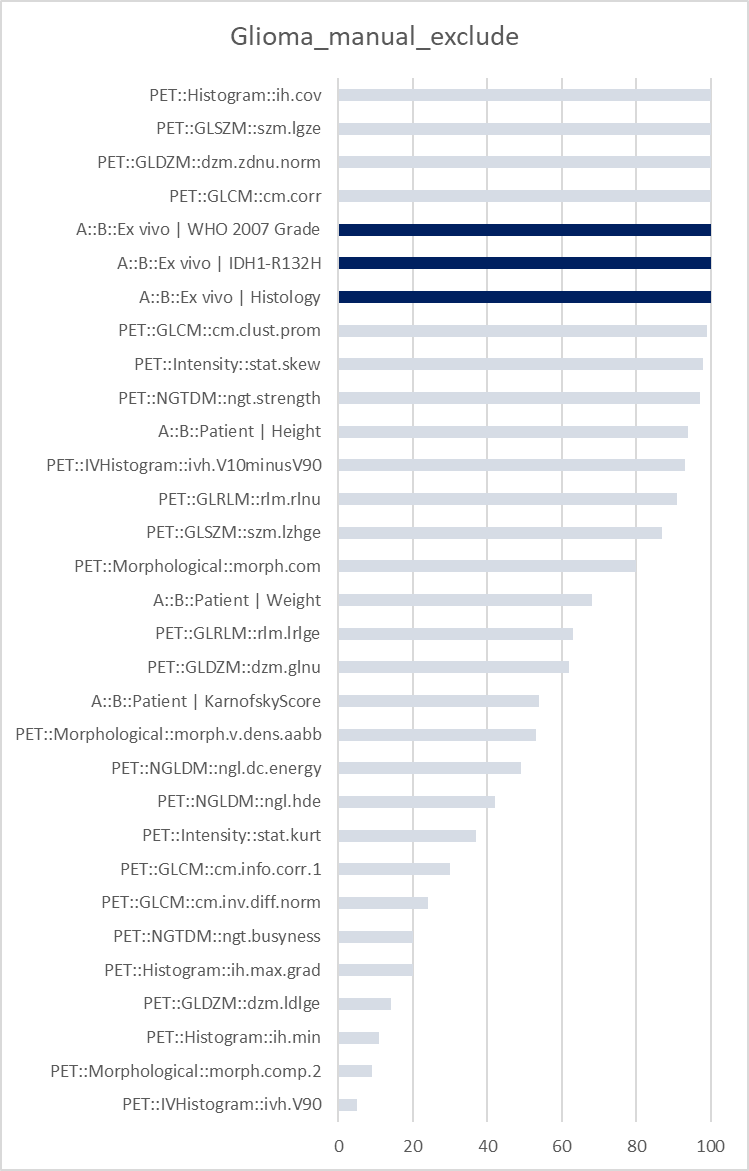


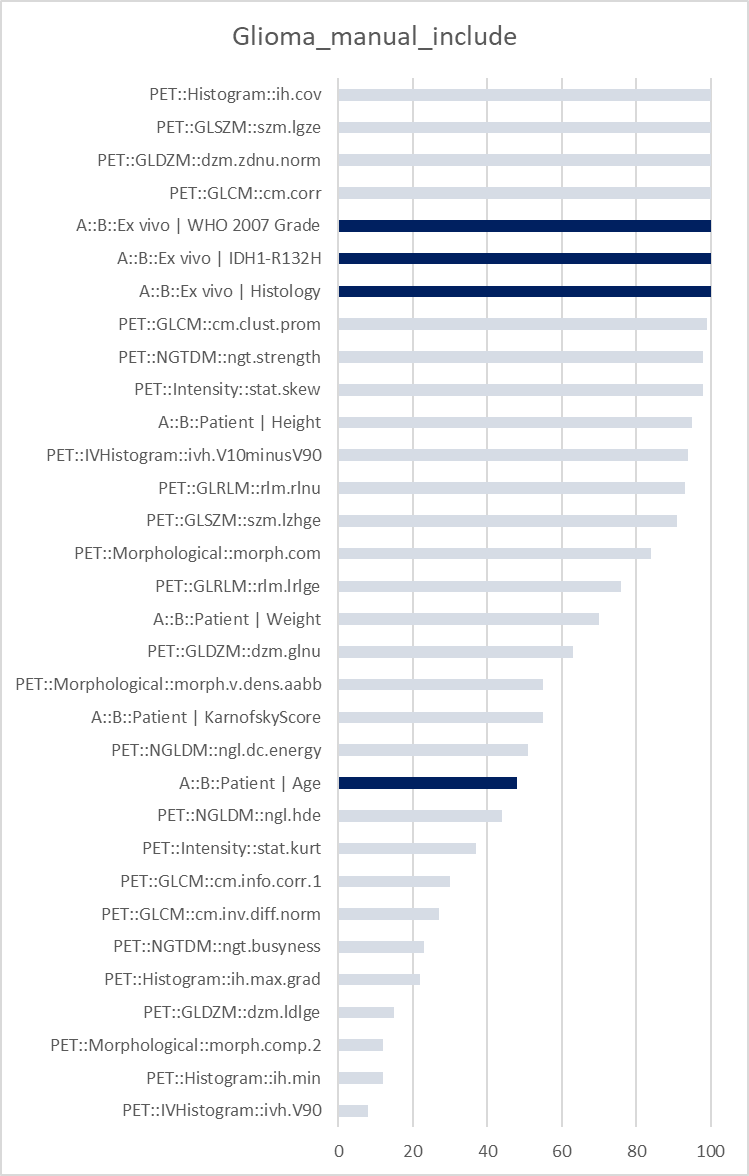

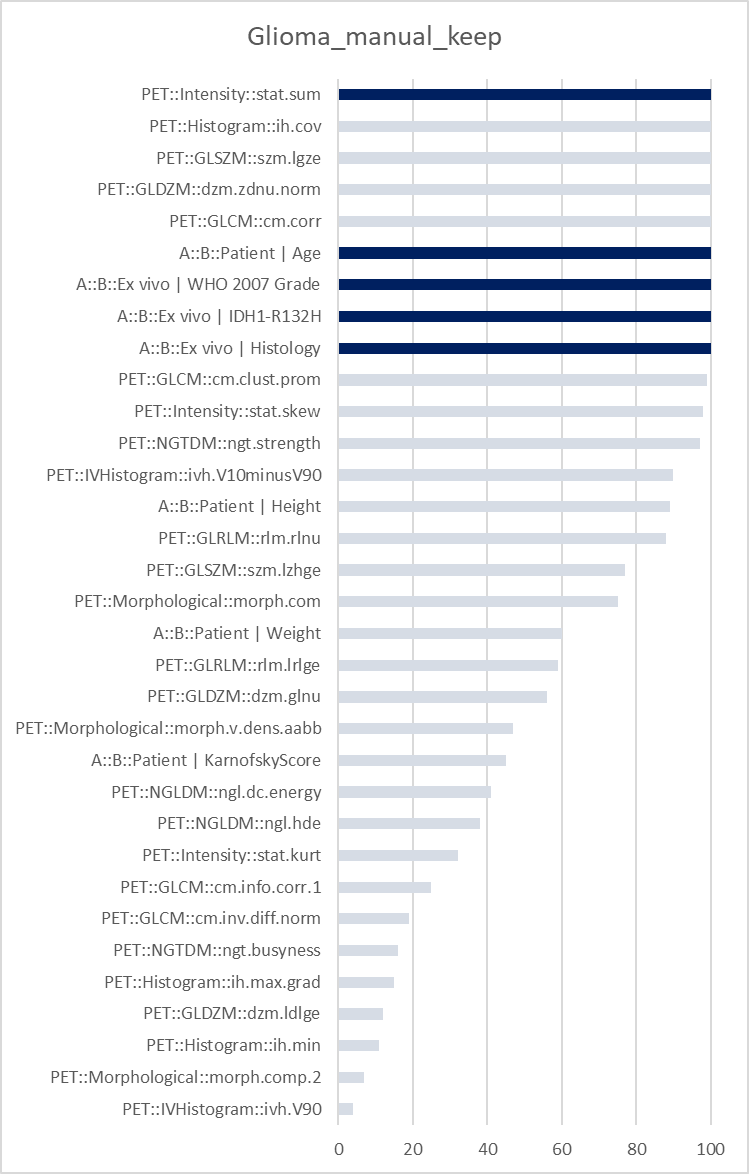


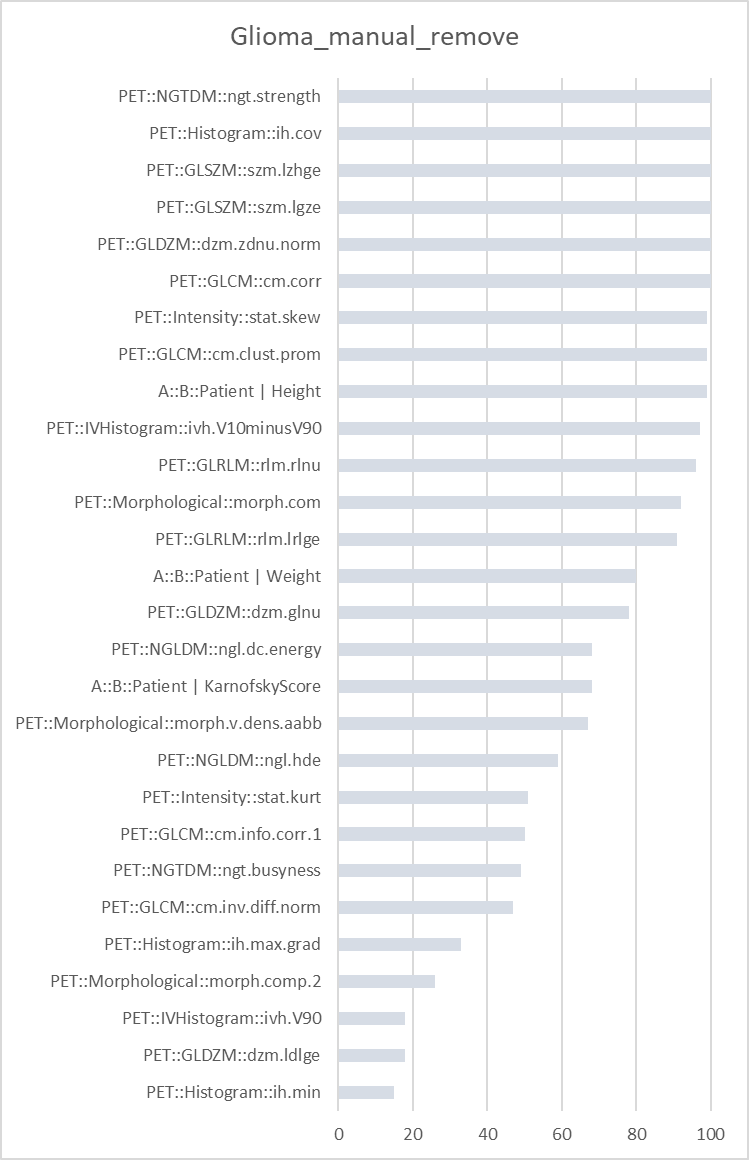


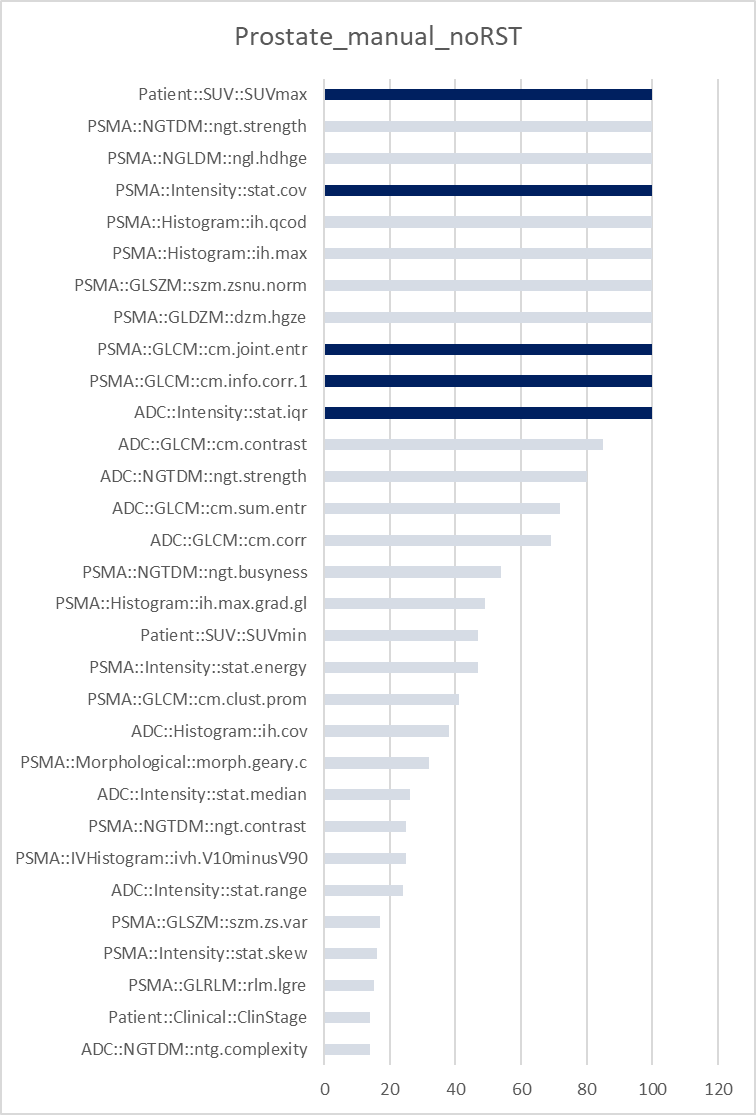

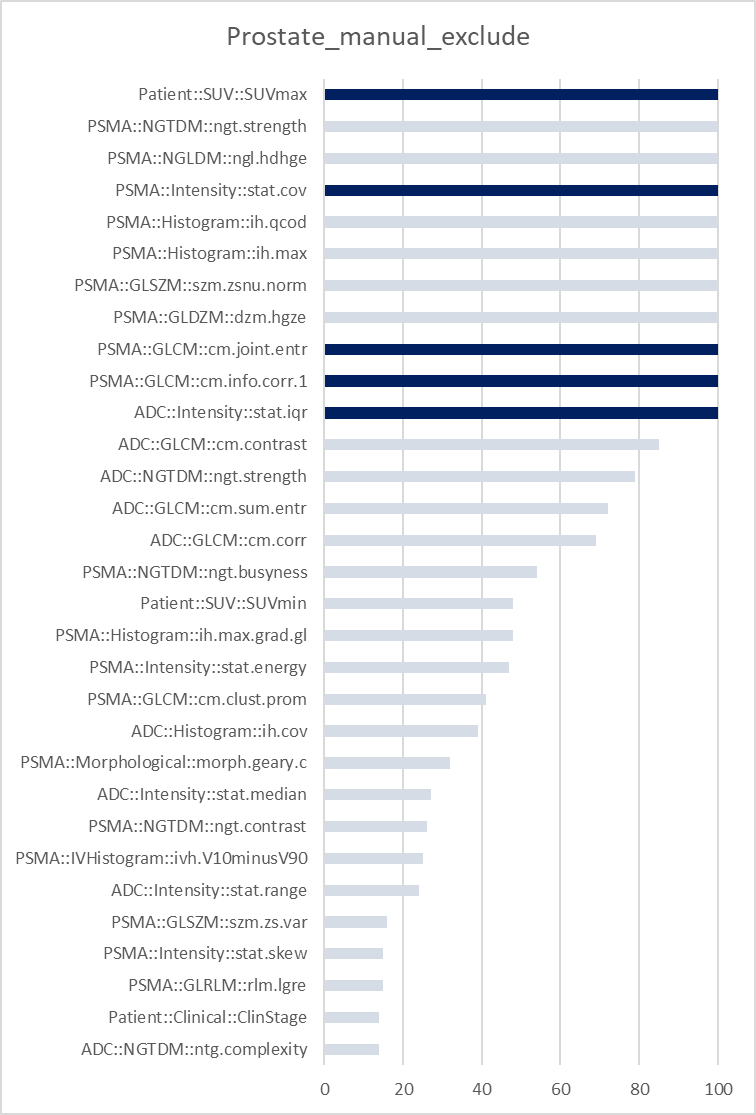


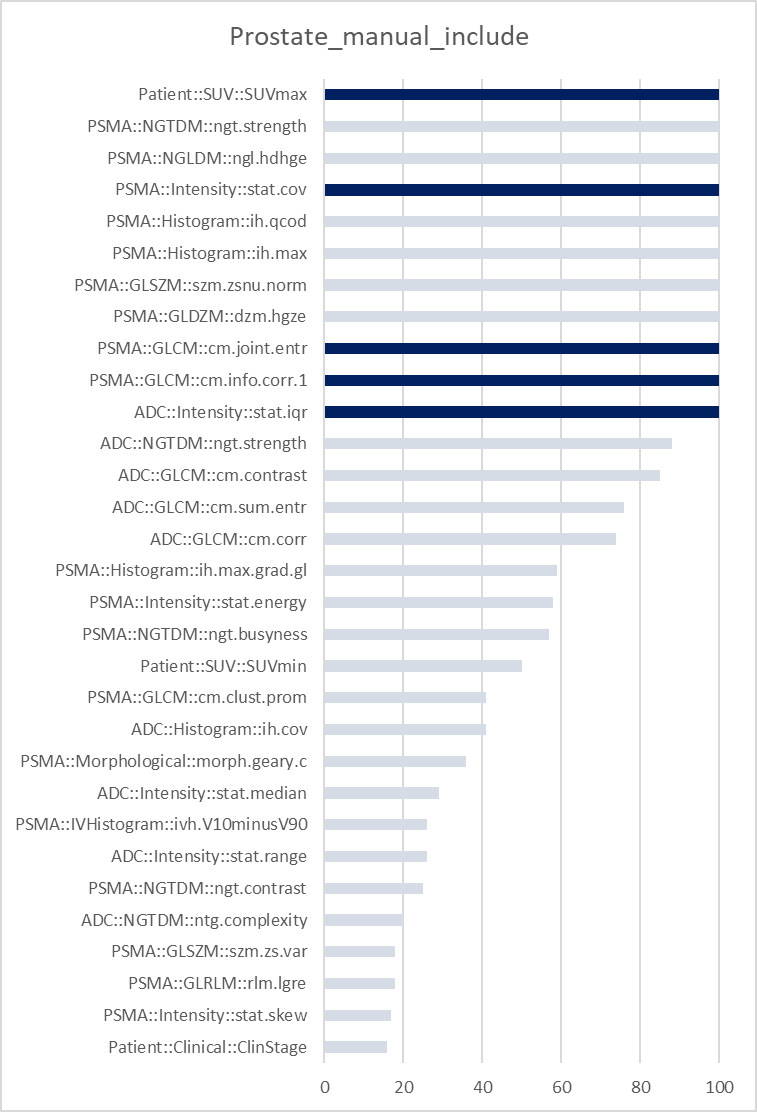

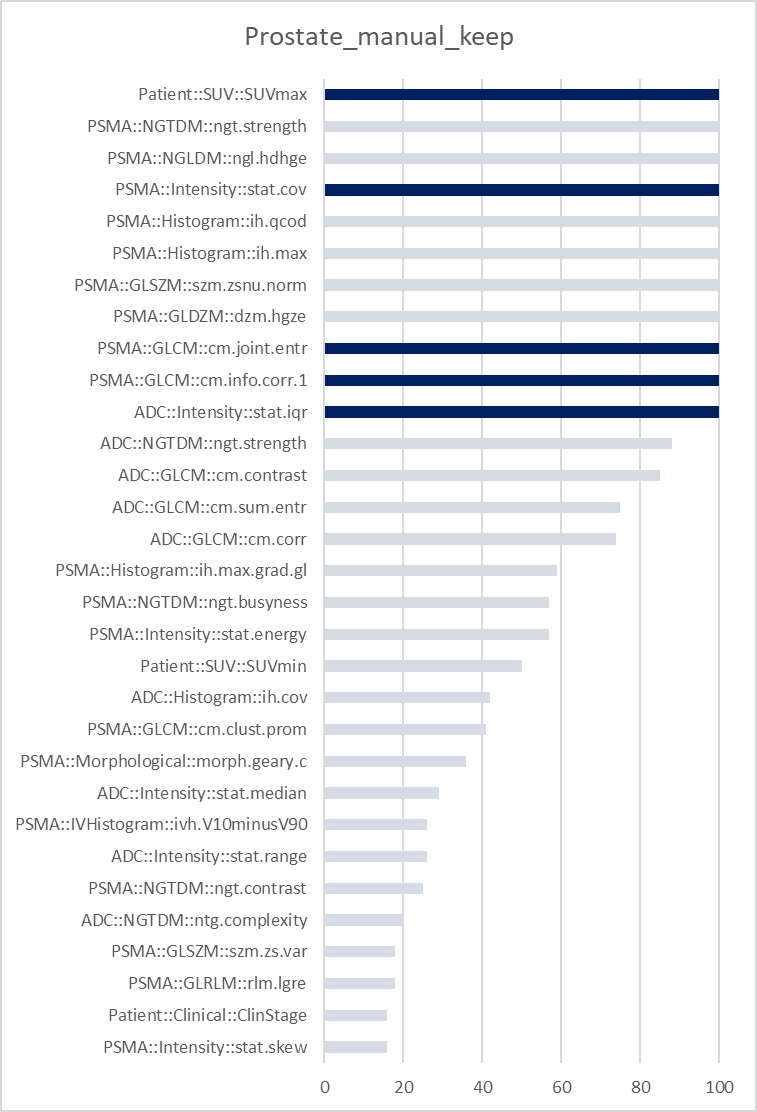


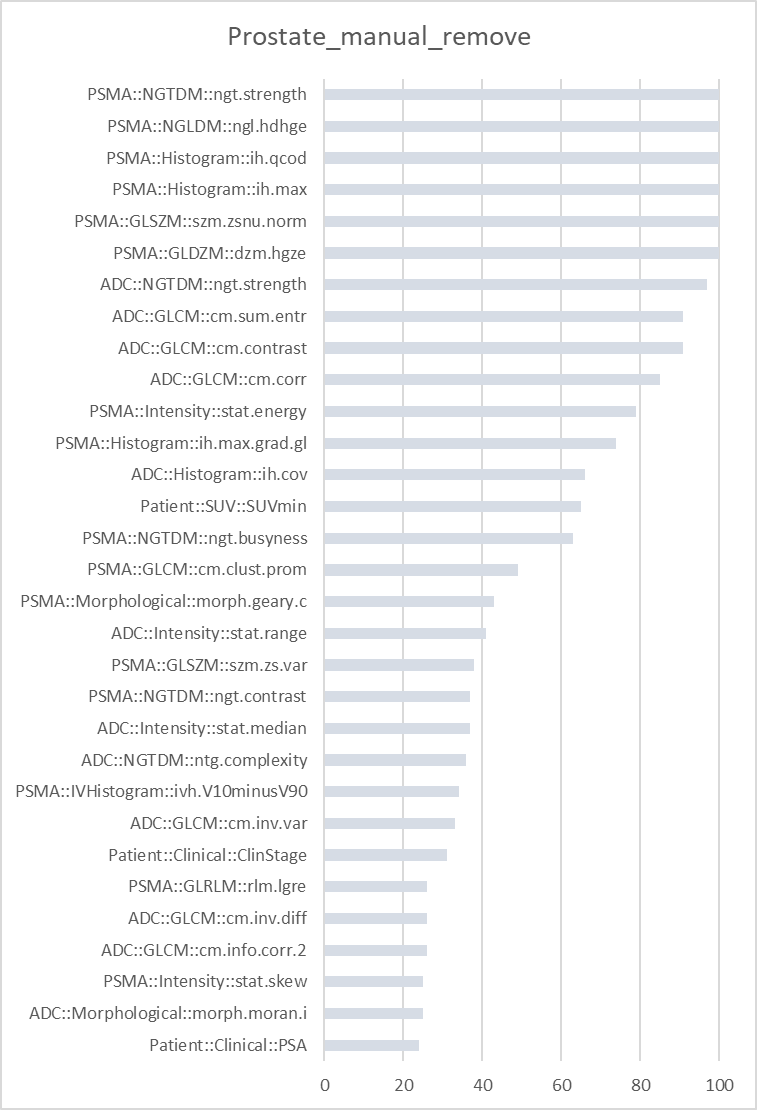


**Supplemental S7: Performance evaluations across all established models**

Confusion matrix-based performance evaluation of all established models in both MLDP and manual preprocessing scenarios. The study utilized the Extreme gradient boosting (XGBoost) learning scheme for ML model building.

|  |  | noRST | | remove | | keep | | include | | exclude | |
| --- | --- | --- | --- | --- | --- | --- | --- | --- | --- | --- | --- |
| Data | Metric | MLDP | Manual | MLDP | Manual | MLDP | Manual | MLDP | Manual | MLDP | Manual |
| DLBCL | ACC | 0,82 | 0,77 | 0,52 | 0,68 | 0,82 | 0,77 | 0,77 | 0,80 | 0,73 | 0,73 |
|  | SNS | 0,79 | 0,71 | 0,57 | 0,00 | 0,79 | 0,71 | 0,79 | 0,79 | 0,43 | 0,71 |
|  | SPC | 0,83 | 0,80 | 0,50 | 1,00 | 0,83 | 0,80 | 0,77 | 0,80 | 0,87 | 0,73 |
|  | NPV | 0,89 | 0,86 | 0,71 | 0,68 | 0,89 | 0,86 | 0,88 | 0,89 | 0,76 | 0,85 |
|  | PPV | 0,69 | 0,63 | 0,35 | nan | 0,69 | 0,63 | 0,61 | 0,65 | 0,60 | 0,56 |
| Glioma | ACC | 0,80 | 0,63 | 0,65 | 0,62 | 0,80 | 0,81 | 0,79 | 0,61 | 0,79 | 0,62 |
|  | SNS | 0,85 | 0,72 | 0,81 | 0,72 | 0,85 | 0,87 | 0,83 | 0,72 | 0,85 | 0,71 |
|  | SPC | 0,75 | 0,53 | 0,48 | 0,53 | 0,74 | 0,75 | 0,75 | 0,51 | 0,74 | 0,53 |
|  | NPV | 0,83 | 0,66 | 0,72 | 0,65 | 0,84 | 0,85 | 0,82 | 0,64 | 0,83 | 0,65 |
|  | PPV | 0,77 | 0,60 | 0,60 | 0,60 | 0,77 | 0,77 | 0,77 | 0,59 | 0,77 | 0,60 |
| Prostate | ACC | 0,79 | 0,71 | 0,67 | 0,71 | 0,79 | 0,77 | 0,79 | 0,71 | 0,78 | 0,71 |
|  | SNS | 0,76 | 0,69 | 0,54 | 0,71 | 0,76 | 0,73 | 0,76 | 0,69 | 0,76 | 0,71 |
|  | SPC | 0,81 | 0,73 | 0,79 | 0,70 | 0,82 | 0,82 | 0,81 | 0,73 | 0,80 | 0,71 |
|  | NPV | 0,79 | 0,73 | 0,66 | 0,73 | 0,80 | 0,77 | 0,80 | 0,73 | 0,80 | 0,74 |
|  | PPV | 0,78 | 0,69 | 0,69 | 0,67 | 0,79 | 0,77 | 0,78 | 0,69 | 0,77 | 0,68 |

RST – Rule set table; MLDP – ML-driven data preparation; DLBCL – diffuse large B-cell lymphoma; ACC – accuracy; SNS – Sensitivity; SPC – Specificity; PPV – Positive Predictive Value; NPV – Negative Predictive Value;

**Supplemental S8: CLAIM: Checklist for Artificial Intelligence in Medical Imaging**

| Section / Topic | No. | Item |  |
| --- | --- | --- | --- |
| TITLE / ABSTRACT |  |  |  |
|  | **1** | Identification as a study of AI methodology, specifying the category of technology used (e.g., deep learning) | **Comparison of data preprocessing approaches for radiomic AI studies** |
|  | **2** | Structured summary of study design, methods, results, and conclusions | **See "Abstract" in manuscript.** |
| INTRODUCTION |  |  |  |
|  | **3** | Scientific and clinical background, including the intended use and clinical role of the AI approach | **Investigation of a novel rule set table (RST) mechanism which can give control to clinicians over the process of data preprocessing. See "Introduction" in manuscript for details.** |
|  | **4** | Study objectives and hypotheses | **Compare RST with state-of-the-art data preprocessing methods in radiomic studies** |
| METHODS |  |  |  |
| *Study Design* | **5** | Prospective or retrospective study | **Retrospective study** |
|  | **6** | Study goal, such as model creation, exploratory study, feasibility study, non-inferiority trial | **Feasibility and comparison study.** |
| *Data* | **7** | Data sources | **See “Data access” chapter** |
|  | **8** | Eligibility criteria: how, where, and when potentially eligible participants or studies were identified (e.g., symptoms, results from previous tests, inclusion in registry, patient-care setting, location, dates) | **See CONSORT (Figure 1).** |
|  | **9** | Data pre-processing steps | **See “Data preprocessing pipelines” chapter** |
|  | **10** | Selection of data subsets, if applicable | **NA** |
|  | **11** | Definitions of data elements, with references to Common Data Elements | **See "Cohorts" chapter** |
|  | **12** | De-identification methods | **Data already anonymized as stored in open-access repositories: See “Data access” chapter** |
|  | **13** | How missing data were handled | **No missing data was present** |
| *Ground Truth* | **14** | Definition of ground truth reference standard, in sufficient detail to allow replication | **See references to original papers in "Cohorts" chapter** |
|  | **15** | Rationale for choosing the reference standard (if alternatives exist) | **Clinical relevance** |
|  | **16** | Source of ground-truth annotations; qualifications and preparation of annotators | **See references to original papers in "Cohorts" chapter** |
|  | **17** | Annotation tools | **See references to original papers in "Cohorts" chapter** |
|  | **18** | Measurement of inter- and intrarater variability; methods to mitigate variability and/or resolve discrepancies | **See references to original papers in "Cohorts" chapter** |
| *Data Partitions* | **19** | Intended sample size and how it was determined | **See references to original papers in "Cohorts" chapter** |
|  | **20** | How data were assigned to partitions; specify proportions | **See "** **Performance Evaluation" chapter in manuscript** |
|  | **21** | Level at which partitions are disjoint (e.g., image, study, patient, institution) | **No same patient data was allowed to be present in one train-test split across the cross-validation colds** |
| *Model* | **22** | Detailed description of model, including inputs, outputs, all intermediate layers and connections | **See "Methods" in manuscript and Supplemental** |
|  | **23** | Software libraries, frameworks, and packages | **See "Methods" in manuscript and Supplemental** |
|  | **24** | Initialization of model parameters (e.g., randomization, transfer learning) | **See Supplemental for details** |
| *Training* | **25** | Details of training approach, including data augmentation, hyperparameters, number of models trained | **See "Methods" in manuscript and Supplemental** |
|  | **26** | Method of selecting the final model | **No final model was selected. Study compared multiple data preprocessing methods** |
|  | **27** | Ensembling techniques, if applicable | **NA** |
| *Evaluation* | **28** | Metrics of model performance | **Confusion matrix analytics in test set** |
|  | **29** | Statistical measures of significance and uncertainty (e.g., confidence intervals) | **Confidence intervals (CI) of test predictive performances with 95% confidence levels**  **ANOVA test with p<0.05 significance threshold** |
|  | **30** | Robustness or sensitivity analysis | **All confusion metrics including sensitivity, specificity, positive and negative predictive values, accuracy and balanced accuracy were measured only in test subsets of the cross-validation and the independent test schemes** |
|  | **31** | Methods for explainability or interpretability (e.g., saliency maps), and how they were validated | **NA** |
|  | **32** | Validation or testing on external data | **Cross-validation with train-test splits of a single-center cohort. In case of dual-center cohort, one center was used for training and other for testing. Only test predictive performance values were reported** |
| RESULTS |  |  |  |
| *Data* | **33** | Flow of participants or cases, using a diagram to indicate inclusion and exclusion | **NA** |
|  | **34** | Demographic and clinical characteristics of cases in each partition | **NA** |
| *Model performance* | **35** | Performance metrics for optimal model(s) on all data partitions | **Confusion matrix performance metrics in test subsets** |
|  | **36** | Estimates of diagnostic accuracy and their precision (such as 95% confidence intervals) | **Average test performance metrics across 100-folds in case of cross-validation. 95% CI for each performance metric in case of cross-validation** |
|  | **37** | Failure analysis of incorrectly classified cases | **NA** |
| DISCUSSION |  |  |  |
|  | **38** | Study limitations, including potential bias, statistical uncertainty, and generalizability | **See "Discussion" chapter in manuscript for details** |
|  | **39** | Implications for practice, including the intended use and/or clinical role | **Control can be given to clinicians over data preprocessing of radiomic studies** |
| OTHER INFORMATION |  |  |  |
|  | **40** | Registration number and name of registry | **See references to original papers in "Cohorts" chapter** |
|  | **41** | Where the full study protocol can be accessed | **See references to original papers in "Cohorts" chapter** |
|  | **42** | Sources of funding and other support; role of funders | **Medical University of Vienna-funded project** |

Mongan J, Moy L, Kahn CE Jr. Checklist for Artificial Intelligence in Medical Imaging (CLAIM): a guide for authors and reviewers. Radiol Artif Intell 2020; 2(2):e200029. <https://doi.org/10.1148/ryai.2020200029>

**Supplemental S9: Rules template example for RST input**

Example of an RST module-supported rules to influence the data preprocessing procedures. Any relation (> or <) denotes relations in-between feature values. Note that the actual syntax is to be followed by S1. Any text with courier new refers to the actual syntax.

| **ID** | **Definition** |
| --- | --- |
| 1 | Keep features (A#B#C) |
| 2 | Remove features (A#B#C) |
| 3 | Include features (A#B#C) |
| 4 | Exclude features (A#B#C) |
| 5 | If features (A#B#C) > then feature (X)# then remove |
| 6 | If feature (A) < then (X)# then keep |
| 7 | If feature (A) has more missing# than feature (B)# then keep |
| 8 | If feature (A) has more missing# than feature (B)# then remove |
| 9 | If feature (A) value is(9)then features (B#C#D) must be in range# of (10-11)# else remove |
| 10 | If feature (A#B) are redundant# then keep (S) |

**Supplemental S10: ML predictive performance of various classical ML methods**

Performance of machine learning models trained with Supported vector machine (SVM) and Random forest (RF) classifiers across all cohorts. MLDP – ML-driven data preprocessing; exp – explicit; pref – preferred; DLBCL - Diffuse large B cell lymphoma; ML – machine learning;

| Data | ML algorithm | Preprocessing setup | noRST | exp-remove | exp-keep | pref-keep | pref-remove |
| --- | --- | --- | --- | --- | --- | --- | --- |
| DLBCL | SVM | MLDP | 0.77 | 0.52 | 0.79 | 0.76 | 0.69 |
|  |  | Manual | 0.70 | 0.51 | 0.74 | 0.71 | 0.70 |
|  | RF | MLDP | 0.78 | 0.56 | 0.78 | 0.79 | 0.72 |
|  |  | Manual | 0.75 | 0.54 | 0.72 | 0.76 | 0.69 |
| Glioma | SVM | MLDP | 0.72 | 0.61 | 0.74 | 0.71 | 0.73 |
|  |  | Manual | 0.71 | 0.56 | 0.72 | 0.69 | 0.71 |
|  | RF | MLDP | 0.77 | 0.65 | 0.76 | 0.78 | 0.79 |
|  |  | Manual | 0.69 | 0.63 | 0.71 | 0.69 | 0.65 |
| Prostate | SVM | MLDP | 0.75 | 0.68 | 0.77 | 0.70 | 0.77 |
|  |  | Manual | 0.70 | 0.61 | 0.75 | 0.68 | 0.76 |
|  | RF | MLDP | 0.78 | 0.70 | 0.80 | 0.79 | 0.79 |
|  |  | Manual | 0.73 | 0.66 | 0.78 | 0.78 | 0.77 |

**Supplemental S11: Common ML-related terminology**

| **Term** | **Definition** |
| --- | --- |
| Training data | The set of data consists of multiple samples (patients) to train the ML models. |
| Testing data | Independent dataset to give an objective evaluation of a final ML model |
| Data split | The partitioning of a dataset into different subsets (e.g. training and testing). |
| Classifier | A type of machine learning algorithm used to assign a class label to a data input. |
| Cross-validation | A technique used in machine learning to evaluate the performance of a model on unseen data by creating multiple folds or subsets. |
| Pipeline of algorithms | A sequentially ordered set of algorithms |
| Hyperparameters | Configuration variables of an algorithm which serve to fine-tune the behaviour of a given algorithm |
| Feature extraction | A process which involves identifying and extracting the most relevant features from an image |
| Feature selection | A process of determining the most relevant features for model construction. |
| Feature ranking | A procedure that measures the contributions of individual features (variables) to the performance of an ML model |
